# Advancing Single-Cell Transcriptomic Analysis to Reveal Age-Related Skeletal Muscle Changes: A Systematic Review

**DOI:** 10.14336/AD.2025.0701

**Published:** 2026-09-03

**Authors:** Chaoran Liu, Hei Yuet Wong, Ranyang Tao, Baoqi Li, Pui Yan Wong, Chun Ho Tam, Ning Zhang, Ling Qin, Jiankun Xu, Gustavo Duque, Christoph Brochhausen, Wing Hoi Cheung, Ronald Man Yeung Wong

**Affiliations:** ^1^Department of Orthopaedics & Traumatology, The Chinese University of Hong Kong, Hong Kong, China; ^2^Bone, Muscle & Geroscience Research Group, Research Institute of the McGill University Health Centre, Montreal, Canada.; ^3^Institute of Pathology, University Medical Center Mannheim, Heidelberg University, Mannheim, Germany.

**Keywords:** Muscle, Sarcopenia, Aging, Single-cell, Single-nuclei, Systematic review

## Abstract

Population aging has become a widespread health problem that leads to huge socioeconomic burden. Skeletal muscle as an important component of motor system, gradually degenerates with age. Age-related muscle disorders, such as sarcopenia is associated with higher risks of falls, fracture, disability, and mortality in old people. As there is still no Food and Drug Administration (FDA) approved drug to treat sarcopenia, conducting research of in-depth mechanisms is warranted to develop novel treatments. The cutting-edge techniques single-cell and single-nuclei RNA sequencing can help to address this issue by discovering age-related changes of muscle at the single-cell level. This review aims to systematically explore current evidence of age-related muscle changes during normal aging, regeneration, and after treatments at the single-cell level. 29 studies were eligible and included in the current review according to the PRISMA guideline. The muscle cell composition was altered with age, such as diminished muscle stem cells (MuSCs), vascular cells, Schwann cells, and increased myocytes as well as some types of immune cells. Inflammation levels, collagen and extracellular matrix (ECM) signaling, protein catabolism, TGFβ signaling, apoptosis, and autophagy of MuSCs, myocytes, fibro-adipogenic progenitor cells, vascular cells, or immune cells were regulated with age. Delayed muscle regeneration of aged muscle was relied on disorders of cell-specific immune response, myogenesis, angiogenesis, and ECM remodeling. Three treatments involved in this review could reverse age-related dysfunction of muscle cells to some extent. Further research targeting age-related changes of muscle at the single-cell level is an important tool in assisting development of more effective treatments for sarcopenia.

## INTRODUCTION

Aging is associated with a gradual loss of skeletal muscle mass, which declines from 50% of total body mass in young adults to 25% in older people [1]. Sarcopenia is an age-related muscle disorder, characterized by progressive loss of muscle mass, strength, and poor physical performance [2]. In individuals aged 65 years and older, the prevalence of sarcopenia ranges from 10% to 40%. This condition is related to increased risks of falls, fractures, disability, and mortality [3-5]. Although malnutrition, inactivity, and chronic diseases may cause sarcopenia, the exact mechanism remains unclear. Additionally, there is no Food and Drug Administration (FDA) approved drug for this disorder [2]. Resistance exercise and special dietary patterns can delay the loss of muscle mass and strength, but more appropriate treatments are warranted due to the poor adherence of old people [6]. Human skeletal muscle bulk-RNA sequencing exhibit significant differences between young and aged muscle in cellular senescence, insulin signaling, myogenesis, oxidative phosphorylation, and adipogenesis [7]. Targeting these alterations of muscle can be an approach to reverse sarcopenia. Nevertheless, it is not the optimal strategy, as different cell types in muscle play distinct roles in maintaining muscle homeostasis. Age-related changes may also vary amongst different cell types [1].

With the development of next-generation sequencing for musculoskeletal conditions [8, 9], the particular changes of skeletal muscle at cellular levels can be detected by single-cell (scRNA-seq) or single-nucleus RNA sequencing (snRNA-seq) [1]. Special analyses such as cell proportion in tissues, gene expression and functional enrichment in specific cell population, subcluster characteristics, differentiation trajectory, cell-cell communication, and RNA velocity can be conducted by these techniques [10]. As for skeletal muscle, upregulated Wnt signaling pathways in muscle stem cells (MuSCs) [11], FOXO signaling in type I and II myofibers, TGFβ pathways in fibro-adipogenic progenitors (FAPs) [1], and pro-inflammatory status in immune cells [12] were found in aged muscle. Interestingly, exercise could reduce the inflammatory response of immune cells in aged muscles [13]. During aged muscle regeneration after injury, impaired immune cell activation and angiogenesis were associated with delayed muscle recovery [14]. The objective of this study is to summarize alterations between young and aged muscle, and age-related discrepancies of muscle regeneration and treatment at cellular levels. Understanding the changes in different muscle cells during the aging process can lead to the development of more targeted treatments for sarcopenia, ultimately reducing mortality and the socio-economic burden amongst the old population.

## MATERIALS AND METHODS

### Search strategy

Literature search was conducted in PubMed, Embase and Web of Science from inception until 26^th^ November 2024. Following Preferred Reporting Items for Systematic Reviews and Meta-Analyses (PRISMA) guidelines, (single-cell OR scRNA OR single-nucleus OR single-nuclei OR sNuc OR snRNA) AND (aging OR ageing) AND (muscle OR sarcopenia) were used as keywords for searching articles in all fields.

### Search criteria

Included articles were based on the following criteria: (1) studies using scRNA or snRNA sequencing; (2) comparison between muscle of young and old people/animals with or without interventions. The exclusion criteria were: (1) methodology studies; (2) non-English papers; (3) conference abstract; (4) studies without peer-review.

### Study selection

Two authors (CL and YHW) independently screened all titles and abstracts of searched papers after removing duplicates. The full text of the remaining papers was identified according to the inclusion and exclusion criteria. Any discrepancies were discussed and finally determined by a third reviewer (RMYW).

### Data extraction

The following data were extracted by two reviewers (CL and YHW): author name, publication year, species, age, sample size, sequencing methods, cell type, marker genes of cells. Differential cell population, gene expression and pathways of cells, other scRNA-seq or snRNA-seq specific analyses, and special subcluster characteristics were also described. Qualitative analysis was conducted for all studies included in this review. The risk of bias (RoB) of clinical studies was analyzed by Cochrane RoB tool, and animal studies by SYRCLE's RoB tool [15].

## RESULTS

A total of 1,396 studies were extracted from the three databases. After removal of duplicates, 1,078 studies were screened, and 29 were finally included (Fig. 1). 17 studies compared characteristics of muscle cell between young and old people or animals [1, 11, 12, 16-29]. 8 studies showed the differences in muscle regeneration between aged and young animals [14, 30-36], and 3 of them also had comparison with age-related normal muscle. Treatments were performed in 4 studies [13, 37-39], and these studies have the untreated part of age-related comparisons. The analyses of RoB showed that only 5 studies had items (< 3) with high risk (Fig. 2). RoB details of all studies were shown in Supplementary Figure 1.

### Methodology of included studies

21 studies used scRNA-seq (2 only utilized public datasets), and 10 studies used snRNA-seq. Mice, rats, monkeys, and human were involved in 21, 2, 1, and 6 studies, respectively. Tibialis anterior was the most used animal muscle for sequencing, whilst vastus lateralis was the most used in human. 2 studies contained not only muscle tissue, but also other tissue samples (blood and bone) for sequencing together [17, 24]. All types of muscle cells, MuSCs, macrophages, and CD45^+^ cells were loaded in 21, 5, 1, and 2 studies, respectively. 10× Genomics Chromium system and Illumina platforms are the most commonly used equipment. Details of each study method were shown in Supplementary Table 1. The study designs of all studies were shown in Supplementary Tables 2-4.

**Figure 1.**
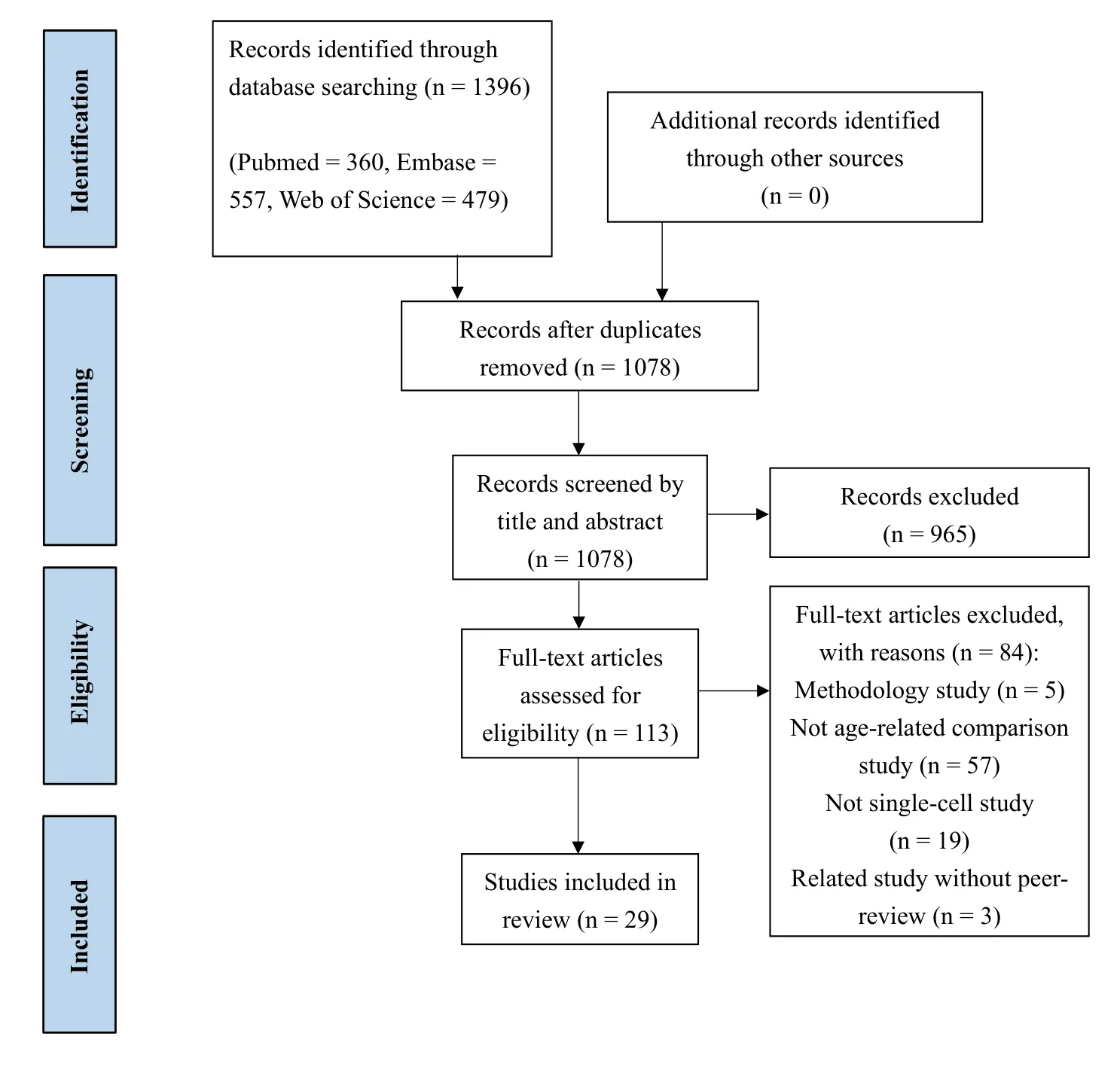
Flow chart of selection process for paper review.

### Age-related changes of muscle cell population

In skeletal muscle, muscle cell types comprise myocytes (type I and II, and mixed fibers), MuSCs/muscle progenitor cells/myoblasts, FAP cells, neuromuscular junction (NMJ), myotendinous junction, and mesenchymal stem cells. The microenvironment of muscle contains endothelial cells (ECs), adipocytes, pericytes, neurons, tenocytes, myeloid, Schwann cells, tendon fibroblasts, fibroblasts, smooth muscle cells (SMCs), and immune cells such as neutrophils, monocytes, macrophages, dendritic cells (DCs), mast cells, and lymphoid cells (T cells, B cells, and NK cells). Marker genes of different cell types were shown in Supplementary Table 5. The proportion of some specific cell types to the total number of muscle cells changed with age. Compared to young mice, old mice had reduced MuSCs, type IIB myonuclei, Schwann cells, FAPs, ECs [13, 16, 19, 21, 22, 29], increased myocytes, and several immune cells [21, 22, 35]. Reduced ECs, FAPs and MuSCs/satellite cells were also found in aged monkeys and rats [11, 38]. In human muscle, old people had reduced satellite cells, ECs/capillary ECs, SMCs, Schwann cells, type IIX and hybrid IIX/IIA myofibers, DCs, pericytes, and increased myocytes, type I myonuclei, adipocytes, venous ECs, and some immune cells [1, 12, 17, 18, 20, 27]. The general age-related changes of cell proportion were presented (Fig. 3).

**Figure 2.**
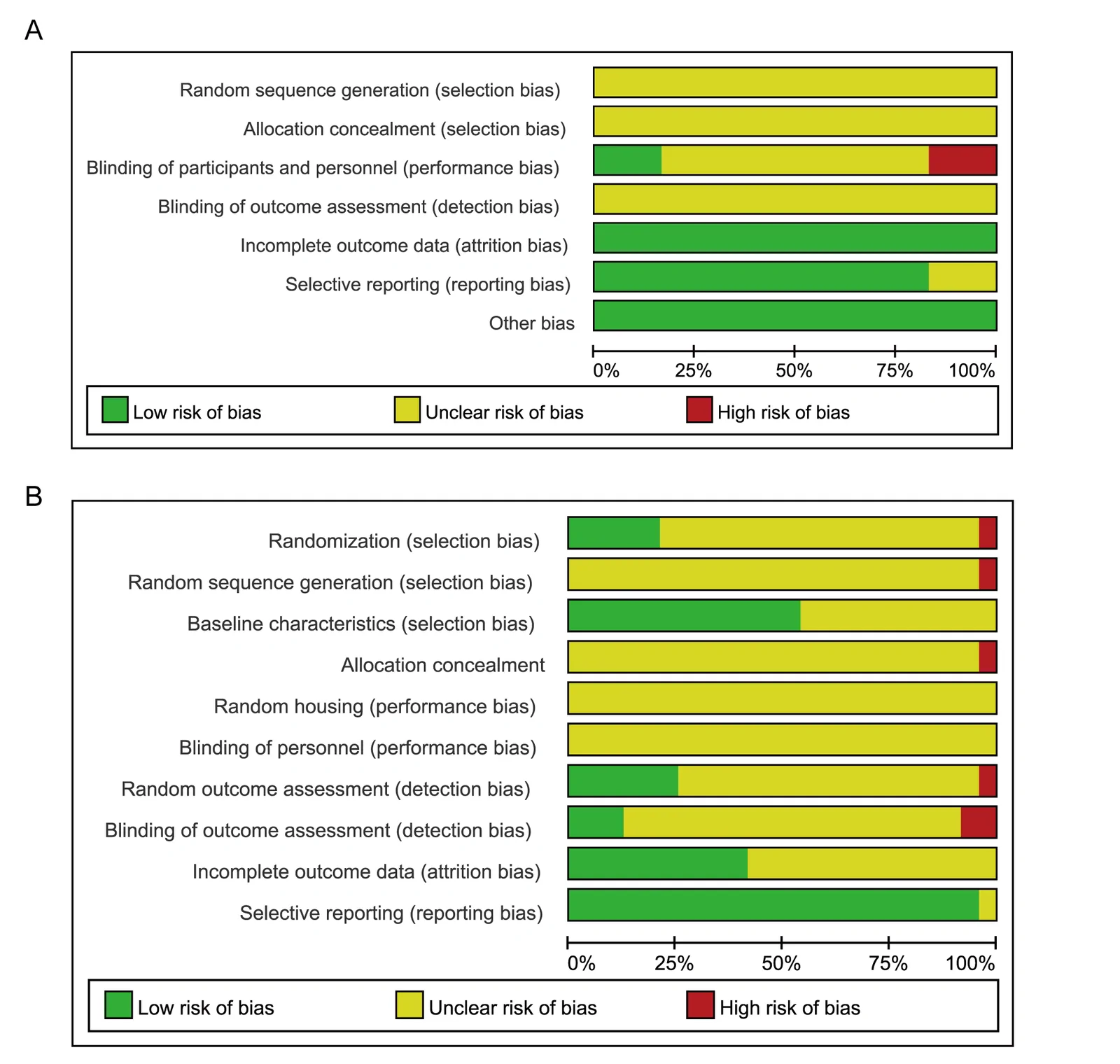
**Risk of bias (RoB) of included studies**. (**A**) summary of clinical studies based on Cochrane RoB tool. (**B**) summary of animal studies based on SYRCLE’s RoB tool.

### Muscle stem cells

MuSCs, also known as “satellite cells”, have the capacity of muscle regeneration. Aged human and mouse MuSCs had higher transcriptional and epigenetic heterogeneity, indicating lower similarity between cells [1, 26]. However, 1 study reported that aged human MuSCs had lower transcriptional heterogeneity [27]. In MuSCs of young and old mice, myogenic factors *Pax7*, *Myod*, *Myf5* have similar expression. Cell quiescence sustainer *Spry1* and cell cycle regulators were down-regulated in aged MuSCs [26]. Collagen and matrix (ECM)-related genes showed significantly increased variability and reduced activity in aged mice [24, 26]. Aged human MuSCs had downregulated collagen and ECM pathways [20]. *Foxo3* expressions were upregulated in young monkey MuSCs, whilst genes *Igf1* and *Vegfa* were increased in aged human and monkey MuSCs, respectively [11, 27]. Chemokine expression, cytokine-mediated signaling, and response to cytokine stimulus were upregulated with age in mice [24, 26]. Upregulated Wnt signaling pathway, response to hypoxia, and downregulated regulations of MAPK cascade, Notch signaling pathway, unfolded protein response were detected in aged MuSCs [11, 24, 27]. Most aged human MuSCs showed higher expression of mitochondrial genes and oxidative phosphorylation (OXPHOS) [1, 20]. There are some subclusters of MuSCs. Commonly, MuSCs can be divided into quiescent and activated status. Although 1 animal study found preserved myogenic differentiation trajectory and fate in aged MuSCs, aberrant transcriptional kinetics were also observed, leading to slower rate of activation compared to young cells [24]. Gene expression of aged mouse cells in the quiescent state was more variable but was reversed in the activated state [25]. Impaired protein folding and cellular response to environmental stress, and decreased FOXO signaling were found in aged quiescent MuSCs, whilst catabolic processes were downregulated and stress responses were upregulated in aged activated MuSCs [1, 25]. Early activated MuSCs that enriched genes related to inflammation, cell growth and autophagy were increased with age in people [1]. Different from total MuSCs, *Myod1* and *Myog* were decreased in aged human cycling MuSCs and muscle progenitors that highly expressed *Cdkn2a* and *Cdkn1c* [27]. Muscle cell differentiation, and interferon gamma were enriched, but ECM and cell matrix adhesion were downregulated in aged muscle progenitor cells [27]. MuSCs subsets that related to glycolysis and mitochondrial respiration were reduced in aged mouse muscle [13]. Higher proportion of CD47^hi^ MuSCs that are related to poor regeneration capacity was found in aged mouse muscle [28]. Aged human subcluster ICA^+^ MuSCs had increased pro-inflammatory gene expression of *Ccl2* [12]. NMJ-related Pax7^+^/S100b^+^ MuSCs were enriched in aged mice. Sod1^-/-^ mice, which is a model of neuromuscular degeneration, had reduced Pax7^+^S100b^-^ MuSCs, and higher expression of genes related to perturbation of stem cell differentiation, and lower expression of genes related to quiescence and healthy MuSCs [32]. MuSCs in aged muscle showed higher inflammation levels, downregulated collagen and ECM related pathways, upregulated OXPHOS, but similar myogenic factors.

**Figure 3.**
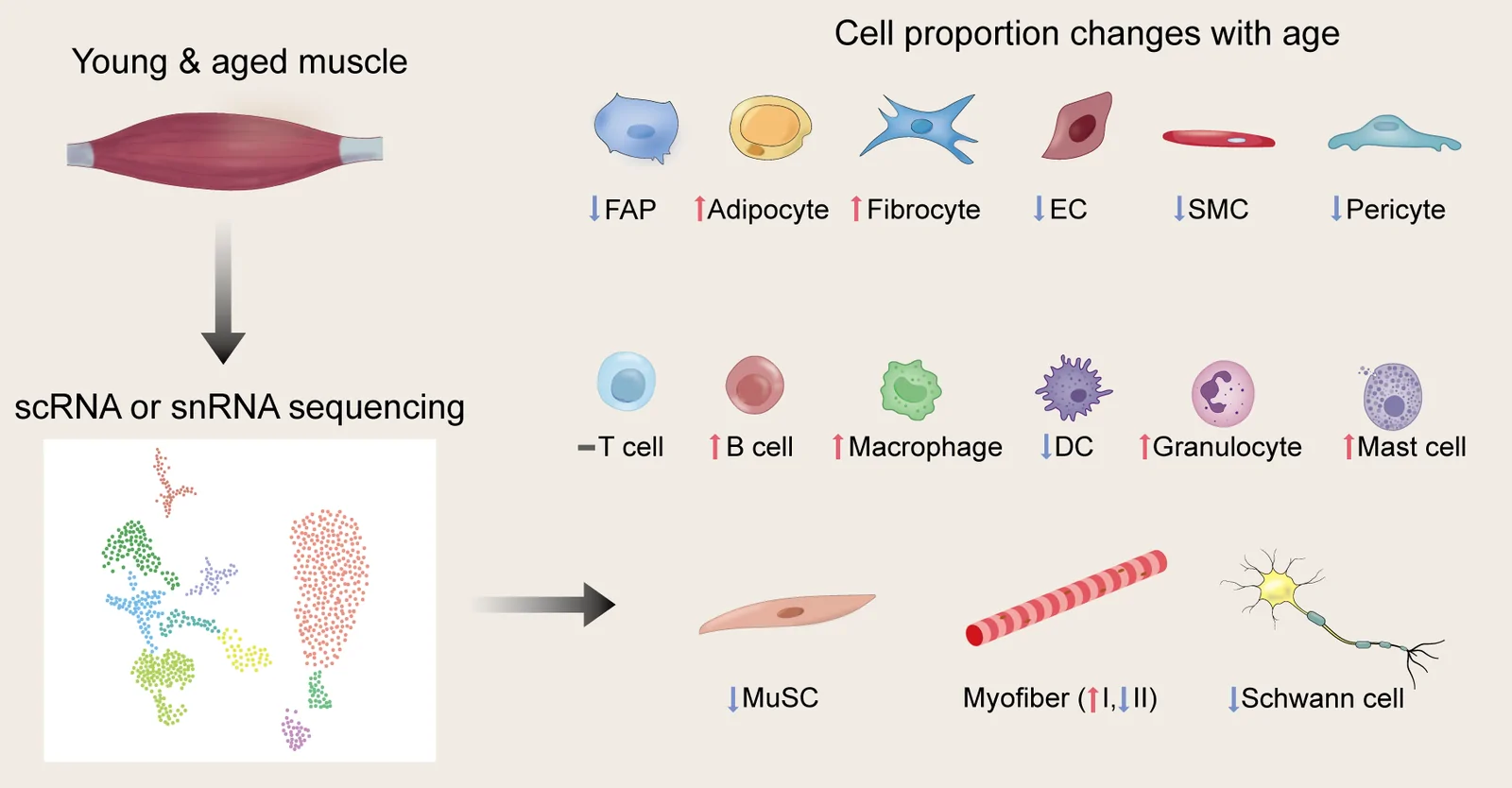
**Age-related changes of cell proportion by scRNA or snRNA sequencing**. Generally reduced MuSCs, type II myofiber, Schwann cells, endothelial cells, SMCs, pericytes, dendritic cells, FAPs, increased type I myofiber, B cells, macrophages/monocytes, granulocytes, mast cells, adipocytes, fibrocytes, and unclear change of T cells were shown in aged than young muscle. Red arrows represented the cell proportion increased with age, blue arrows represented decreased with age, and the black horizontal bar represented unclear change with age.

### Different types of myocytes

Skeletal muscles had different cell types, including fast type IIX, fast type IIA, fast type IIB, slow type I, and hybrid types. There were more fragments in muscle fibers of older individuals [12], as well as lower expression of *Foxo3* and *Sesn1* in aged monkey muscle types [11]. Increased denervation and stress related transcription factors (TFs) were found in both I and II fibers [1]. Downregulations of glucose metabolic process, TFs related to lipid metabolism, upregulation of protein catabolism, FOXO signaling, TGFβ, TNFα signaling pathways, and dysregulated clock genes were shown in aged human I and II myonucleus [1]. According to more abrupt trajectory in type II fibers than type I fibers, they were more sensitive to aging, such as more sharply increased inflammasome, autophagy, and oxidative stress response in type II than type I myonuclei [1, 20, 38]. In aged human type II fiber, decreased Wnt9a were detected, which may induce NMJ dysfunction [1]. Spatial transcriptomics showed that fast fiber-related *Tnnt3* and *Myh2* were downregulated in aged human muscle [20]. Fiber type II has two different isoforms, including IIA and IIX. Increased *Hdac4*, *Trim63*, *Musk*, *Plin2*, and decreased *Vegfa*, *Tnnt3*, *Ppargc1a*, *Foxo3* were found in type IIA fibers [1, 38]. Upregulated response to muscle inactivity and regulation of cellular response to stress, and downregulated muscle system process and MAPK signaling pathway were found in aged monkey fast IIA [11]. Downregulation of FOXO signaling pathway and muscle system process, and upregulation of response to muscle inactivity and negative regulation of transport were shown in aged monkey fast IIX [11]. In aged human type I fiber, increased expression of *Vegfa*, *Prkag3*, and decreased *Ppara*, *Bdnf* were found [1]. Slow fiber of aged monkeys had downregulated PPAR signaling pathway and muscle system process, but upregulated negative regulation of cell development and regulation of chemotaxis [11]. Downregulated OXPHOS and fatty acid degradation, as well as upregulated IL-6 signaling and glycolysis was found in type I fibers during ageing trajectory [1]. However, another human study showed aged slow fiber had upregulated muscle contraction pathways [20]. According to spatial transcriptomics, slow fiber-related *Tnnt1* and *Myh7* were upregulated in aged muscle [20]. Nevertheless, reduced *Tnnt1*, *Myh7*, and increased *Runx1*, *Myog*, *Tgfb2*, *Tnfrsf12a* were found in aged mouse type I fibers in another single-cell study [38]. Type II specific ENOX1^+^ myonuclei that related to carbohydrate metabolism was reduced in aged human muscle [1]. SAA2^+^, TNNT2^+^, ID1^+^, and DCLK1^+^ myonuclei were enriched in the aged group. They were associated with denervation, stress and pro-inflammatory status, and proteolysis [1]. FAM189A2^+^ nuclei in rat slow fiber were decreased with age, which may be related to damaged fiber repairment [12]. Two subclusters of slow fiber that related to mitochondrial dysfunction, and active fatty acid metabolism were only detected in aged individuals [17]. Both type I and II muscle showed dysfunctions of protein synthesis and degradation, glucose metabolic process, and upregulated inflammatory pathways. Type II muscle had downregulation of genes related to NMJ function, whilst mitochondria function and fatty acid metabolism of type I muscle were significantly disturbed with age.

### Fibro-adipogenic progenitors

FAPs are a population of muscle stromal cells, which can differentiate into either fibroblasts or adipocytes. Young and aged mouse FAPs showed higher maximum mean discrepancy, indicating this cell type had significant differences with age [24]. Aged FAPs exhibited higher expression of *Ccn2* and *p16* [16, 39], and downregulated gene sets of multiple ECM and collagen, suggesting their composition may be affected [13, 20, 24, 38, 39]. Circadian negative regulation genes, inflammatory pathways, cholesterol homeostasis, steroid biosynthesis, cellular response to hormone stimulus, and TGFβ stimulus were upregulated in aged animal FAPs [11, 13, 37]. Downregulated PI3K-Akt signaling pathway and copper homeostasis were also found in aged monkey FAPs [11]. Aged human FAP subclusters generally showed downregulated cell-cell adhesion and Wnt pathways, and upregulated mitochondrial biogenesis, TGFβ and IL-6 signaling pathways [1]. Fibroblast-like cells which related to fibroblast activation was elevated, and a subcluster MME^+^ FAPs that associated with adipogenic pathways was decreased with age [1]. Animal studies showed that age-related changes were more obvious in FAPs differentiated cells (adipocytes and myofibroblasts) than progenitors. Aged adipocytes expressed higher adipogenesis genes, whilst enriched TGFβ signaling were found in aged myofibroblasts [13, 24]. Lipid transport-related genes were increased in aged stromal cells [38], whilst increased *Il6* and decreased *Igf1* were found in fibroblasts of aged muscle [12]. Aged FAPs are highly associated with upregulated TGFβ signaling, which is a potential target for further aged muscle treatment.

### Neuromuscular junction

Postsynaptic muscle fiber (PMF) is a functional unit of NMJ. ECM organization and sodium ion transmembrane transporter activity were upregulated, whilst glutamatergic synapse and neuron recognition were downregulated in PMF with age. In aged human muscle, NMJ accessory was significantly increased, which contributed to aged-related NMJ re-innervation [12]. Reduced Schwann cells in aged muscle also led to NMJ deterioration [12, 22]. Functional annotation showed that aged monkey terminal Schwann cells had increased tight junction and actin cytoskeleton organization [11]. Neural functional compensation and neural remodeling are observed in aged NMJ at single-cell levels.

### Vascular cells

Skeletal muscle also contains vascular cells, including ECs, SMCs, and pericytes. Aged human vascular cells had upregulated IL-6, AP-1, TGFβ, and autophagy pathways, and downregulated transmembrane transporter activity, which related to impaired vascular integrity [1]. In aged rodent ECs, increased inflammation-related *S100a9*, *S100a8*, *Cebpb*, and *Il1b* [14], and reduced angiogenesis regulation-related genes were found [38]. Upregulation of mitochondrial genes, positive regulation of apoptotic process, and downregulation of collagen and ECM pathways, Ras signaling pathway, and blood vessel morphogenesis were detected in aged human and monkey ECs [11, 20]. Similar to MuSCs, aged ECs also showed higher response to hypoxia [11, 13]. TNF-α signaling, apoptosis, IFNγ responses, p53, IL6, and mTORC1 signaling of ECs were also regulated by age [13]. CCL2^+^ vasculature ECs was increased with age [12]. A subcluster of ECs that highly expressed GBPs genes and enriched cytokine related pathways, were abundant in muscle from sarcopenic mice [21]. Skeletal muscles contain small amounts of SMCs and pericytes with higher *Il6* expression in aged cells [12]. Improved negative regulation of phosphate metabolic process and regulation of cellular response to stress, and downregulated pathways related to collagen and ECM, glucose metabolism, regulation of cell adhesion and CDC42 GTPase cycle were detected in aged SMCs [11, 13, 20]. Aged human pericytes had upregulated mitochondrial translation elongation, downregulated cell junction assembly, and blood vessel morphogenesis [1]. CCL26^+^ pericytes also elevated with age [12]. Increased inflammation and impaired vascular integrity were found in all types of vascular cells. Since vascular cells received less attention than other cells in skeletal muscle, the reverse of their function may help to develop novel treatments.

### Immune cells

Aged immune cells highly expressed inflammatory response-related genes, which was related to chronic inflammation [14]. In old people, an increment of pro-inflammatory markers *Ccl3*, *Ccl4*, *Cxcl8*, *Cxcl3*, *Ccl17*, *Il1β*, *Nlrp3* and a reduction of *Il10* were found in several immune cells [12]. HLA genes, MAPK signaling, gamma interferon responses, complement cascades, and phagocytosis-related pathways such as ECM degradation were upregulated [1, 20]. Nevertheless, the antigen presentation had contrary alterations in aged immune cells reported by 2 studies [1, 20]. Aged human muscle cells had reduced numbers and strength of interactions with immune cells, especially ECs and type II fibers. Additionally, the overall communications amongst immune cells also decreased [18]. Macrophages underwent remarkable changes with aging [24]. *S100a8*, *S100a9*, *Fabp4*, *Fabp5*, *Il1b* mRNA that related to proinflammatory status, senescence markers *Gpnmb* and *Spp1*, and *Igf1* expression were elevated, whilst reduced anti-inflammatory M2-like markers (*Lyve1*, *Folr2*, *Mrc1*) and *Cd44* were found in aged muscle macrophages [11, 20, 23, 29]. Studies also found genes related to encoding antioxidant enzymes, cell cycle arrest, long-chain fatty acid transporters, lipid metabolic process, inflammation, and positive regulation of chemokine production were highly expressed, whilst those related to chemoattractants, chemotaxis, cellular response to IFNγ, mitochondrial biogenesis, and immune response-regulating signaling pathway had lower expression in aged macrophages [1, 23, 29]. Infiltrating non-classical monocytes, and infiltrating monocyte-derived macrophages were enriched in aged mouse muscle [35]. Studies showed that *Lyve1* was upregulated in aged mice [38], and higher proportion of Lyve1^high^MHC-II^low^ macrophages were observed in aged individuals [18]. However, 2 studies reported that Lyve1^+^ resident macrophages subgroup was reduced, whilst Lyve1^-^ subgroup was abundant in aged mouse muscle [23, 35]. Aged M1 macrophages were associated with upregulated glycolytic and oxidative stress-induced metabolic process [22], and had higher *Cdkn1a* expression [18]. Aged human tissue-resident macrophages (Lyve1^+^ Mrc1^+^) had enhanced cell chemotaxis, regulation of the ERK1/2 cascade, and exerted a pro-angiogenic effect, whilst apoptotic signaling pathway, oxidative stress, and positive regulation of NF-κB transcriptional factor activity were upregulated in aged inflammatory macrophages (Il1β^+^) [17]. Pseudotime analysis is a method used to predict the developmental trajectory of cells based on the single-cell gene expression [33]. Analysis in 1 animal study showed that aged subclusters Lyve1^+^/Mrc1^+^ macrophage was at the beginning, followed by Lyz1^+^ macrophage, and SPP1^+^ and Thbs1^+^ could be found in the whole trajectory, and Gngt2^+^ at the end [29]. Proinflammatory and senescence-related genes were enriched in different subclusters [23]. Elevated inflammatory macrophages (*S1008a*, *S1009a*, *Lrg1*) were found in dexamethasone-injected senescence-accelerated mouse strain P6 (SAMP6) mice [21], which is a well-established sarcopenia model [40]. Subclusters which highly expressed M2-type macrophage markers (likely tissue-resident macrophages), were decreased with age [12, 22]. Another 2 subclusters which contained proinflammatory M1-type macrophage markers were increased with age [22]. Higher expression of SPP1 was found in aged SPP1^+^ macrophages, which was associated with adipogenesis, angiogenesis, OXPHOS, and protein secretion pathways [29]. Cell-cell communication showed unique communication patterns of aged macrophage, such as Spp1-Cd44 receptor-ligands binding pattern [29]. Specific TREM2^+^ macrophages were only found in aged human muscle, which highly expressed *Spp1* and *Nr1h3* [17]. Autophagy decreased in aged human lymphoid cells [1]. Aged mice had increased Tregs, CD8^+^ T cells and γδ T cells were attributed to the expansion of T cells in muscle [35]. Aged T cells had increased genes related to pro-inflammatory activation, cytotoxicity, and exhaustion, and reduced ribosome signaling [13, 35]. Th1- and Th2- like effector memory CD4^+^ T cells were also enriched in aged human muscle, which were related to inflammation and cytotoxicity [17]. Aged mouse B cells showed higher expression of *Cd86* and *Apoe* [35]. Upregulated inflammation-related signaling, ribosome, and downregulated antigen processing and presentation were found in aged mouse B cells [13]. Amongst cell-cell communication between B cells and other immune cells, the highest differential number of interactions of B cells was affecting other B cells, and the stronger interaction between B cells and T/NK cells could be found with aging [35]. Aged neutrophils had upregulated genes associated with aberrant immunoglobulins, chemotaxis and inflammation [35]. In old people, muscle DCs had higher abundance of CD226^+^ and ADAM28^+^ cells, and NK cells showed more NCR1^+^, CD226^+^ and KLRD1^+^ cells [18]. Almost all immune cells were altered with age. Higher inflammatory status and reduction of cell-cell communication in aged immune cells may influence the response after injury and further delay the muscle regeneration.

**Figure 4.**
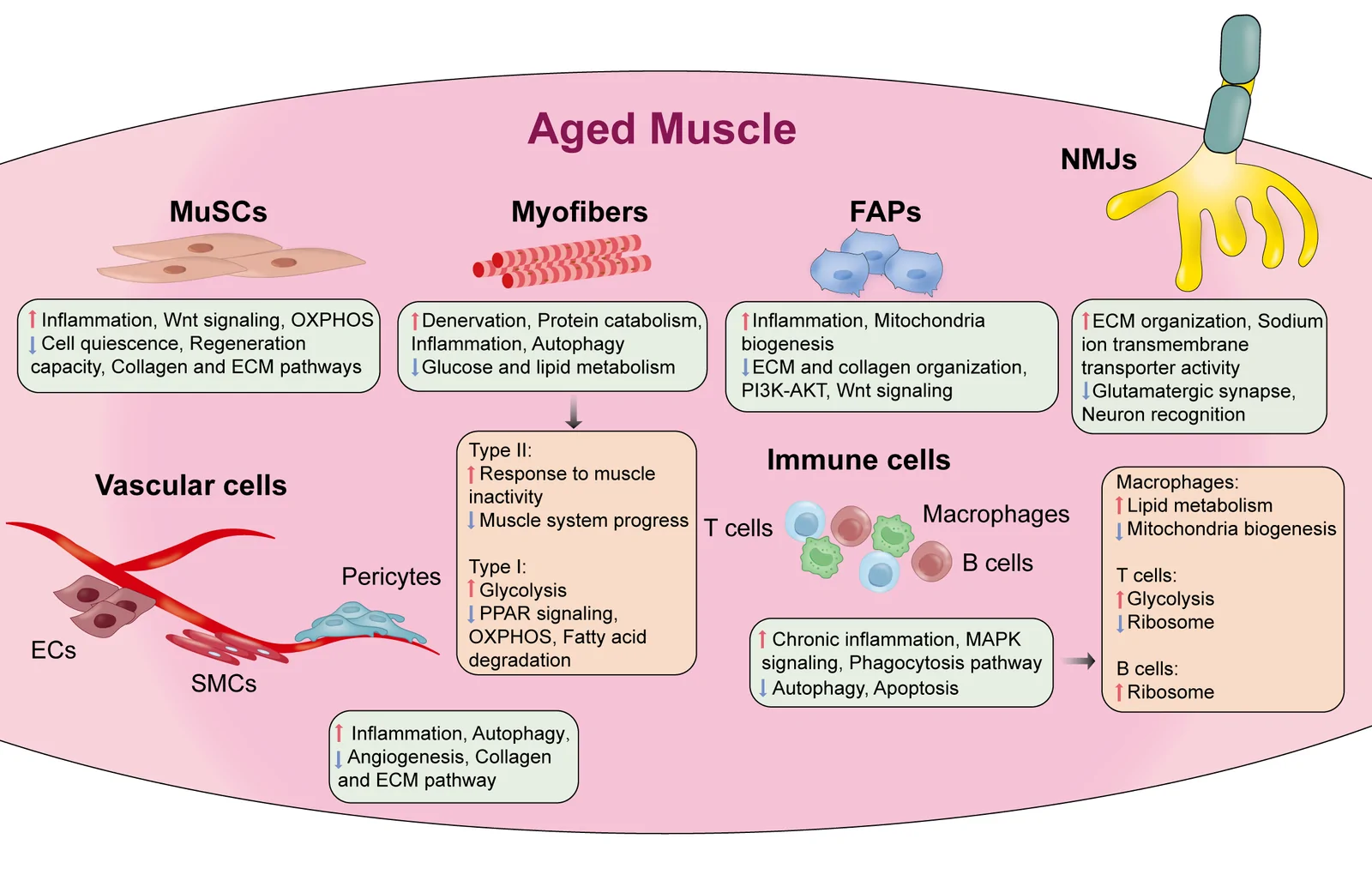
**Functional annotation of aged muscle cells by scRNA or snRNA sequencing analysis**. Representative functions of MuSCs, myofibers (type II and type I), FAPs, NMJs, vascular cells, and immune cells (macrophage, T cells, B cells) in aged muscle compared to young muscle were displayed. Red arrows represented the pathway upregulated with age, blue arrows represented downregulated with age.

### Integrated myocytes and microenvironmental cells alteration

Pseudotime analysis showed that aged mouse myogenic cells had similar trajectory of differentiation compared to young cells, but delayed pseudotime elucidated slow differentiation and lower RNA velocity variance [24]. The pseudotime analysis of another human study showed the starting state enriched in young myocytes. A subcluster in the end point with unique transcript features, and associated with DNA damage response, ubiquitination, and autophagy were more abundant in aged myocytes [17]. Muscle cell-cell interactions were weaker in sarcopenic mice [21]. However, 1 human study found that interaction involving myeloid and lymphoid cells became stronger with age according to 3 main interaction categories: inflammation, ECM, and growth factors [1]. 1 study indicated that the dominate interaction of aged muscle was macrophages and antigen presenting cells, which was different from strong interaction between FAPs and pericytes in young muscle [39]. From old to very old, muscle microenvironmental cells gradually transition from pro-inflammatory to profibrotic status [1]. In most muscle cell types, increased *Pdk4* and decreased *Igfn1*, and circadian clock *Arntl* were found in old people [20, 37]. A *Enah^+^* cluster with *Atf3*, *Flnc*, and *Nrap* as marker genes, was related to development and aging. Muscle *Nr4a3* and *Smad3* expressions were increased in aged mice [19]. Aged muscles from mouse and human shared several similar functional pathways, such as increased phagosome synthesis, antigen processing and presentation, complement and coagulation cascades, and decreased focal adhesion, PI3K-Akt pathway, insulin secretion, and axon guidance [12]. Age-related representative functional changes of muscle at the single-cell level were shown (Fig. 4).

### Age-related cell characteristics during muscle regeneration

Aged muscles have delayed muscle regeneration after injury. This part summarized the mechanisms at single-cell levels based on rodent models in all studies. During the regeneration, age-related alterations of immune cells, MuSCs, FAPs and ECs could interpret the reduction of regenerative capacity in aged muscle. Immune cells including macrophages, NK cells, and eosinophils played important roles in the activation of muscle regeneration. Aged muscle tissue showed higher chronic inflammation and upregulated apoptosis-related genes since injury. In contrast, young muscle had acute inflammatory response at 2 days post-injury (dpi), and negative cytokine regulation was found at 4 dpi, indicating that young immune cells were more easily activated [14]. Hindlimb unloading led to higher inflammation response in aged macrophages. After reuploading, inflammatory response was further elevated in muscle macrophages, but more in young than aged cells, indicating blunted inflammatory transcripts in aged macrophages. Impaired glycolysis pathway was also found in aged macrophages [34]. Mrc1^+^ monocytes/macrophages which responded early to injury, have higher abundance from 2 to 7 dpi in geriatric than young muscle. The peak of Ctsa^+^ patrolling monocytes/macrophages abundance was delayed 1.5 days in geriatric muscle, and NK cells were not increased as noticeably as younger muscle after injury [36]. Repair-associated macrophages that related to growth factors, lipid metabolism, and lysosomal activity were reduced and age-associated macrophages increased in the late stage of regeneration in aged muscle, which was associated with delayed inflammatory response and fibrosis. A significant reduction of eosinophils at 1 dpi, proliferating macrophage population at 3 dpi, and increased lymphoid compartment at 5 dpi were found in aged muscle [35]. The muscle regeneration capacity also depended on MuSCs. The peak abundance of MuSCs and progenitors was different according to age, reflected by peak at 5 dpi in young muscle, 3.5 dpi in old muscle, and 7 dpi in geriatric muscle [36]. During the regeneration of aged muscle injury, Pax7^hi^Myf5^hi^ quiescent-like state MuSCs altered to an activated state after injury, and becoming committed progenitors by 3 dpi, and either differentiating or returning to quiescence by 7 dpi [32]. Different subclusters of MuSCs were dominantly activated in young and aged muscles after injury. The subcluster activated in young muscle was highly related to muscle development, differentiation and cell cycle regulation, whilst those in aged muscle were associated with apoptosis, aging, cellular senescence, and attenuated differentiation ability [14]. Aged muscle after injury had reduced *Myog*, *Myod1*, and *Sox11* expression in MuSCs subsets at 7 dpi [30]. Spatial transcriptomic maps showed that more fusing myocytes and FAPs in the young injury zone, but more monocytes/macrophages, T cells and myonuclei in the aged injury zone [36]. A cluster of slow-activating FAPs that related to negative regulation of cellular proliferation, peptidase activity, cell cycle activity, and upregulated ECM organization, was increased in aged muscle at 4 dpi compared to young muscle [31]. Upregulated ECs migration and angiogenesis were found in young muscle, and negative angiogenesis regulation was shown in aged muscle at 2 dpi [14]. *Cdkn2a* and *Cdkn1a* are senescence hallmark genes. During the regeneration, Cdkn2a^+^ cycling T cells and adipogenic FAPs were increased in aged muscle. Higher abundance of Cdkn2a^+^ Cdkn1a^+^ MuSCs and progenitors were found in aged muscle at 3.5 dpi [36]. Pseudotime analysis identified two branches of myonuclear during muscle regeneration. One branch beginning with MuSCs showed upregulated synapse protein localization and myogenic progenitor fusion in aged muscle, and another branch ending with myogenesis exhibited downregulated OXPHOS and canonical glycolysis disorder [33]. Another pseudotime analysis revealed that a higher fraction of non-G1 (S/G2/M) cells was present in MuSCs in aged muscle [36]. Aged muscle regeneration delayed due to slow inflammatory response of immune cells, impaired myogenesis capacity of MuSCs, slow activation of FAPs, and angiogenesis dysfunction of ECs.

### Age-related muscle cell changes after treatments

Several animal studies reported effects of treatments on aged muscles at the single-cell level, including mechanotherapy, caloric restriction, and exercise. Fibrosis-related and inflammation-related genes were upregulated in FAPs of aged muscle recovered from hindlimb suspension. Mechanotherapy could attenuate these changes compared to weight bearing alone [39]. The receptor-ligand network analysis showed that immune cells interactions were predominate after aged muscle recovery by reloading, which was different from young muscle with the interaction of pericytes and FAPs. Under the mechanotherapy condition, ECs and FAPs were highly interacted in aged muscle. Increments of pentose phosphate, OXPHOS, and ECM regulators were found in aged macrophages after mechanotherapy. Pseudotime analysis of macrophage indicated that mechanotherapy could help reduce the inflammation during aged muscle recovery [39]. Caloric restriction (CR) as a strategy to delay aging, could also benefit muscle. Although CR did not reverse the reduction of satellite cells, 30 genes including cytoskeletal genes *Tnnt1* (slow fiber), *Myh7* (slow fiber), and *Mylk2* (slow fiber, fast fiber IIA and IIX, satellite cells) that reduced in aged muscle were rescued by CR [38]. Exercise could regulate MuSCs subsets that related to glycolysis and immune response [13]. In aged animals, monocytes had the highest number of DEGs after exercise, especially inflammation-related pathways [13, 37]. Age-induced impairment of intercellular communication of Schwann cells and ECs with other cells, were restored by exercise as well [13]. Exercise also upregulated muscle structure development, motor neuron function, OXPHOS, ECM organization, and vasculature development. Interestingly, several circadian clock genes that impaired by aging were rescued after exercise, such as *Arntl* in ECs and FAPs [37]. scRNA-seq showed that aged muscle recovery process became closer to young muscles via mechanotherapy. Caloric restriction only slightly reversed muscle aging. Exercise might be the most useful strategy for sarcopenia, which significantly reduced inflammation levels, mitochondria, neural and vascular function. Due to limited reports of single-cell level results in interventional studies of aged muscle, it is warranted to conduct more for mechanism exploration. Characteristics of aged muscle regeneration and response of treatment were displayed (Fig. 5). All abbreviations were shown in Supplementary Table 6.

**Figure 5.**
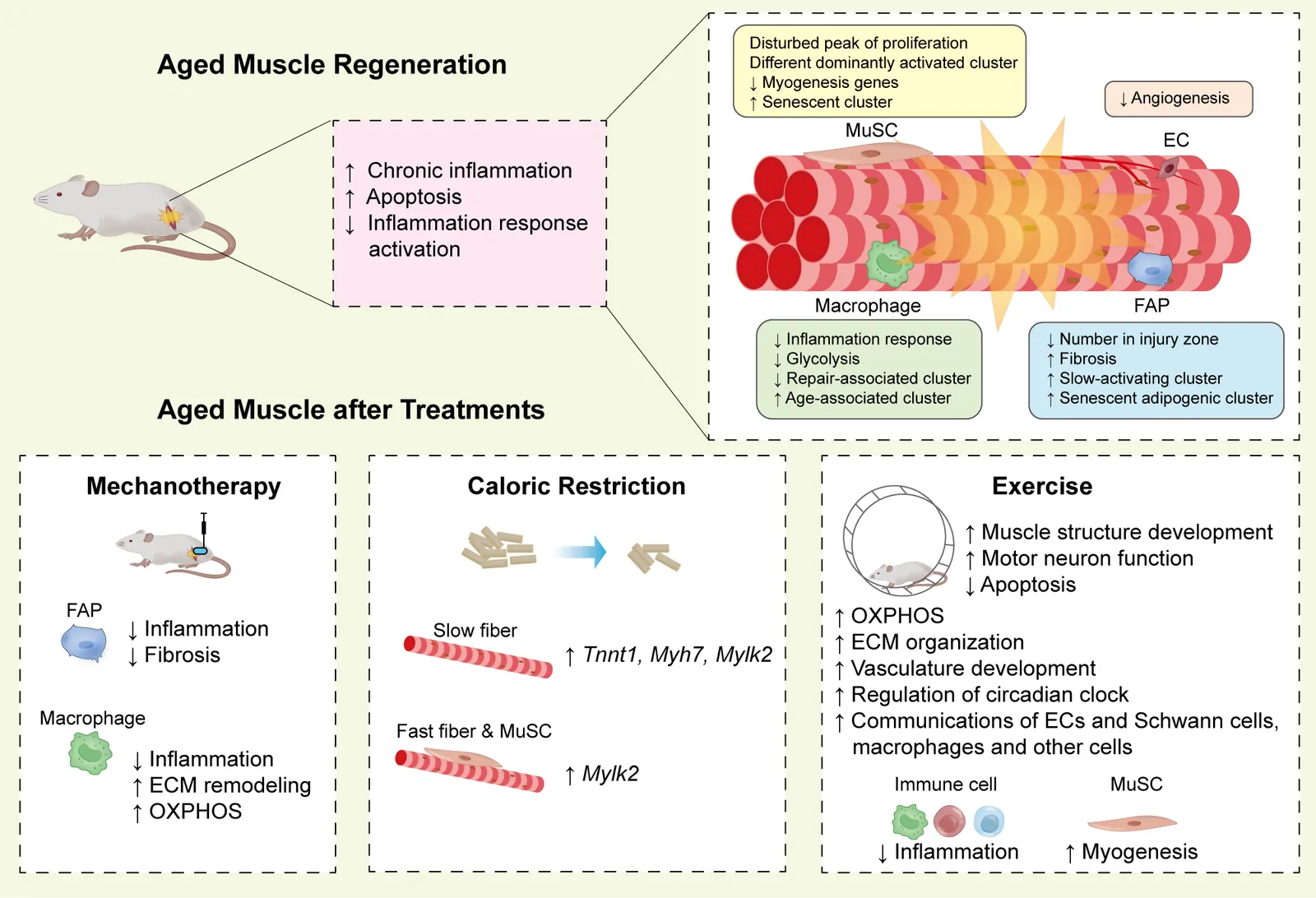
**Regeneration process of aged muscle and response of treatments**. During muscle regeneration after injury or unloading, higher levels of chronic inflammation and apoptosis, and delayed activation of inflammation response were found in aged muscle. Mechanotherapy regulated FAPs and macrophages during muscle recovery from disuse. Caloric restriction significantly rescued alteration of genes with age. Exercise improved immune cells and MuSCs function, and enhanced muscle structure, vessel and neuron function, and metabolism of aged muscle.

## DISCUSSION

Sarcopenia known as an age-related muscle disorder, is a global concern that contributes to a huge socioeconomic burden [41, 42]. New techniques including scRNA-seq and snRNA-seq can help to discover novel mechanisms and develop innovative treatments.

In this review, the composition of skeletal muscle cells altered with age have been summarized. From sequencing results, aged animal or individuals had reduced MuSCs, ECs, FAPs, SMCs, Schwann cells, and increased myocytes. More macrophages in aged mice, whilst more type I myocytes, adipocytes, and most immune cells were elevated in old people. Therefore, higher inflammatory status and lower muscle regeneration capacity in aged muscle could be found. Age-related loss of Schwann cells reduced neuromuscular transmission and led to muscle dysfunction [43].

In contrast to overall changes in gene expression observed in aged muscle, distinct cell types might exhibit unique alterations. Aged MuSCs had elevated transcriptional heterogeneity [1, 26]. Interestingly, different from lower expression of *Igf1* and *Vegfa* in aged muscle [44, 45], aged MuSCs showed higher expression [11, 27], indicating their capacity of ECs recruitment might not be affected. Upregulated genes related to cytokine and chemokine pathways and Wnt signaling was found in aged MuSCs, which may lead to age-related muscle inflammation and fibrosis [46]. However, genes related to collagen and ECM pathways in aged MuSCs had lower expression and activity, and higher variability [20, 24, 26]. The main reason for accumulation of ECM with age may be due to the degradation impairment [47]. Most aged MuSCs subclusters had upregulated OXPHOS [1], which was opposite to age-related changes of total muscle [48]. Since MuSCs activation is associated with anaerobic glycolysis and the differentiation is related to higher OXPHOS [49], lower activation rate and higher cell differentiation were found in aged MuSCs/muscle progenitor cells [12, 24, 27]. The MuSCs decreased subcluster that related to glycolysis was consistent with this finding [13].

Muscle fibers which contain I and II types, were found to be more fragmental in aged muscle [12]. Aged myofiber had upregulated protein catabolic process and cytokine pathways, and downregulations of glucose and lipid metabolism, and muscle contraction. The dysregulation of circadian rhythm was also observed in both aged muscle types [1, 11]. Type I had downregulated OXPHOS, PPAR signaling, and fatty acid degradation, and upregulated IL-6, and impairment of cell development [1, 11]. Similarly, subclusters of slow fiber that related to mitochondrial dysfunction and fatty acid metabolism were abundant with age [17]. Aged type IIA had downregulated MAPK signaling pathways, upregulated autophagy and response to denervation, whilst type IIX had upregulated negative regulation of transport [11]. Mitochondria and fatty acid/lipid metabolism were mainly affected in type I myocyte by age, whilst response to muscle inactivity was upregulated and muscle system progress was downregulated in both aged type IIA and IIX myocytes.

FAPs senescence is a hallmark of muscle aging [24]. Similar to MuSCs, aged FAPs also had downregulations of ECM and collagen [13, 20, 24]. Nevertheless, an increase in fibrotic tissue was found in aged muscle, which may be attributed to elevated TGFβ signaling pathways in both FAPs and myofibroblasts [24, 50]. Upregulated inflammatory pathways, mitochondria biogenesis, and cholesterol homeostasis, as well as downregulated PI3K-Akt and Wnt signaling were found in aged FAPs [1, 11, 13]. Therefore, the treatment that improved mitochondria biogenesis may not benefit FAPs dysfunction. Age-related NMJ degeneration was also detected at single-cell levels, including reduced Schwann cells and dysfunctions of PMF [12, 22].

Muscle microenvironment also includes vascular and immune cells, which are closely related to muscle regeneration. Impaired capillary function, especially increased apoptosis of vascular cells in aged muscle was associated with sarcopenia [51]. Characteristics of aged vascular cells were upregulated pro-inflammatory status, dysfunction of cell junction and transporter activity [1]. Aged immune cells showed higher inflammation levels and phagocytosis [1, 12]. Inflammatory macrophages were increased in sarcopenic muscle [21], and related to higher apoptosis, oxidative stress, and inflammatory response [17]. Upregulated lipid metabolic process, and downregulated mitochondrial biogenesis were found in aged macrophages [29]. Both aged T and B cells exhibited higher inflammation levels, and aged neutrophils, DCs, and NK cells also had alterations related to immune response [13, 18, 35]. Even with a lower number, immune cells played important roles in muscle homeostasis.

Age-related inflammation elevation was found in almost all cell types in muscle. To further distinguish the differences of old people with or without sarcopenia, a study identified that subclusters of FAPs, ECs, and MuSCs contributed to pro-inflammatory status, fibrosis, and glucose uptake inhibition of sarcopenia [52]. Although remarkable mitochondria dysfunction in old compared to young people could be found, inflammation and fibrosis of muscle were hallmarks of sarcopenia in old people. Recent studies supported that autophagy is an anti-aging process of skeletal muscle [53], however, both myofiber types especially type II fiber, vascular cells, and a subcluster of MuSCs exhibited increased autophagy [1]. Due to the discrepancies of whole muscle tissue and specific muscle cells, new sarcopenia treatments should be developed based on changes at the cellular level. Decreased autophagy was only found in B, T, NK, and NKT cells [1]. Single-cell results showed that aged muscle had lower transcription rates, positive pseudotime interactions with mitochondria respiration genes, and different dominant cell-cell communication such as macrophages and antigen presenting cells [24, 39].

Aging complicates muscle injury recovery due to dysfunctions of MuSCs, FAPs, ECs, macrophages, and other immune cells [54]. Previous studies elucidated that aged muscle during regeneration had reduced pro-inflammatory macrophages, declined regenerative potential of MuSCs, and inflammatory and fibrotic microenvironment induced by FAPs [55-57]. The regeneration process is quite different from quiescent condition of aged muscle, especially macrophage subclusters. At the early-stage post injury, inflammatory response was significantly lower in aged muscle [14, 34]. Ctsa^+^ patrolling monocytes/macrophages which should respond earlier, delayed with age [36]. Lower abundance of repair-associated macrophages was found in aged muscle at the late stage [35]. Activated MuSCs in aged muscle exhibited higher levels of apoptosis and cellular senescence [14]. Myogenic capacity of aged MuSCs was also impaired [30]. The slow-activating FAPs which had higher abundance in aged muscle at 4 dpi, were associated with reduced cellular proliferation and increased ECM secretion [31]. Aged ECs had impaired angiogenic capacity which may also be attributed to delayed muscle recovery [14].

During the muscle recovery, mechanotherapy could attenuate fibrosis and inflammation of aged FAPs, upregulate ECM remodeling as well as reduce inflammation of aged macrophages [39]. Significant reductions in proteoglycan and collagen after mechanotherapy improved muscle contraction in aged atrophic muscle, which has been proven by immunostaining [39]. CR as a dietary treatment, could increase lifespan and satellite cell number of aged muscle [58, 59]. The scRNA-seq analysis also revealed that CR could reverse age-related changes of genes mainly in slow fiber, fast fiber, and satellite cells [38]. Since CR reduced both body mass and muscle mass [58], this treatment was not recommended for sarcopenia. Exercise could regulate MuSCs subsets that related to glycolysis and immune response, reduce inflammation levels of muscle cells, improve motor neuron and vascular function, OXPHOS, and ECM organization in aged animal muscle [13, 37]. In human scRNA-seq studies, exercise accelerated myogenic progenitor cells towards maturation [60]. Further studies focusing on effects of resistant exercises on aged muscle at single-cell levels is warranted.

Overall, cell proportion changes could be the apparent senescent biomarkers. Upregulated Wnt signaling, inflammation, OXPHOS, and downregulated collagen and ECM pathways were remarkable features of aged MuSCs. Upregulated protein catabolism, autophagy, and inflammation of both muscle types, FAPs and NMJ dysfunction, higher inflammation in immune and vascular cells can also be diagnostic indicators or biomarkers of age-related muscle disorders. Reversing these changes may help delay sarcopenia. The disturbed cell components, immune environment, myogenesis, angiogenesis, ECM remodeling, delayed activation of MuSCs and FAPs of aged muscle during regeneration were main causes of delayed muscle healing. Further research and development of drugs can focus on novel mechanisms for recovery of aged muscle injury. Potential treatments for sarcopenia requiring further research include modulation of the gut microbiota [61, 62], vibration therapy that can provide mechanical stimuli [63, 64], and senotherapeutics [65].

There are several strengths and limitations of this study. This is the first review that systematically summarized the age-related changes of muscle at single-cell levels. Normal muscles with aging, age-related muscle regeneration process, and aged muscle alterations after treatments have been reported. However, the study mainly showed results based on the single-cell transcriptomic analysis, instead of single-cell proteomics and verified experiments using other methods, such as flow cytometer, western blotting, quantitative polymerase chain reaction, animal or cell models of specific genotypes. Technical variability between studies including sequencing platforms, tissue sources, bioinformatics pipelines increased the study heterogeneity, which may cover up some consistent results. Future research should focus more on the specific muscle changes in sarcopenia at the single-cell level by using advanced platforms to develop more effective treatments.

## Conclusions

In this systematic review, we revealed the composition changes of muscle cells with age, age-related gene expression and function changes of different muscle cell types, and response of aged muscle to regeneration and treatment based on scRNA- or snRNA-seq methods. Lower abundance of MuSCs, ECs, Schwann cells, and more myocytes were found in both old animals and humans. Inflammation levels were generally elevated in all muscle cell types. Upregulated OXPHOS and downregulated collagen and ECM signaling in MuSCs, higher protein catabolism in type I and II myocytes, upregulated TGFβ signaling in FAPs, increased apoptosis in vascular cells, dysfunction of synapse and neuron in NMJ, and obvious pro-inflammatory status in immune cells were characteristics of aged muscle. During aged muscle regeneration, the delayed process was associated with impaired immune response, myogenesis, angiogenesis, and ECM remodeling of aged immune cells, MuSCs, FAPs, and ECs. Treatments including mechanotherapy, CR, and exercise demonstrated benefits in aged muscle through attenuating inflammation levels, improving neuron and vascular functions, and ECM organization. Based on the in-depth mechanisms at the single-cell level, developing new therapies for age-related muscle diseases, such as sarcopenia is warranted.

## Supplementary Materials

The Supplementary data can be found online at: www.aginganddisease.org/EN/10.14336/AD.2025.0701.
