# Supplementary material for: Advancing Single-Cell Transcriptomic Analysis to Reveal Age-Related Skeletal Muscle Changes: A Systematic Review
Source: Aging Dis. 2026 Sep 3;17(6):3195–209. doi: 10.14336/AD.2025.0701 (PMC13653692; doi:10.14336/AD.2025.0701)
Supplement: Supplementary file 1 [file AD-17-6-3195-s.pdf]

SUPPLEMENTARY DATA

Advancing Single-Cell Transcriptomic Analysis to Reveal Age-Related Skeletal Muscle Changes: A Systematic Review

Chaoran Liu, Hei Yuet Wong, Ranyang Tao, Baoqi Li, Pui Yan Wong, Chun Ho Tam, Ning Zhang, Ling Qin, Jiankun Xu, Gustavo Duque, Christoph Brochhausen, Wing Hoi Cheung, Ronald Man Yeung Wong

© 2025. Liu C et al. Published online at http://www.aginganddisease.org/EN/10.14336/AD.2025.0701

SUPPLEMENTARY DATA

Supporting information

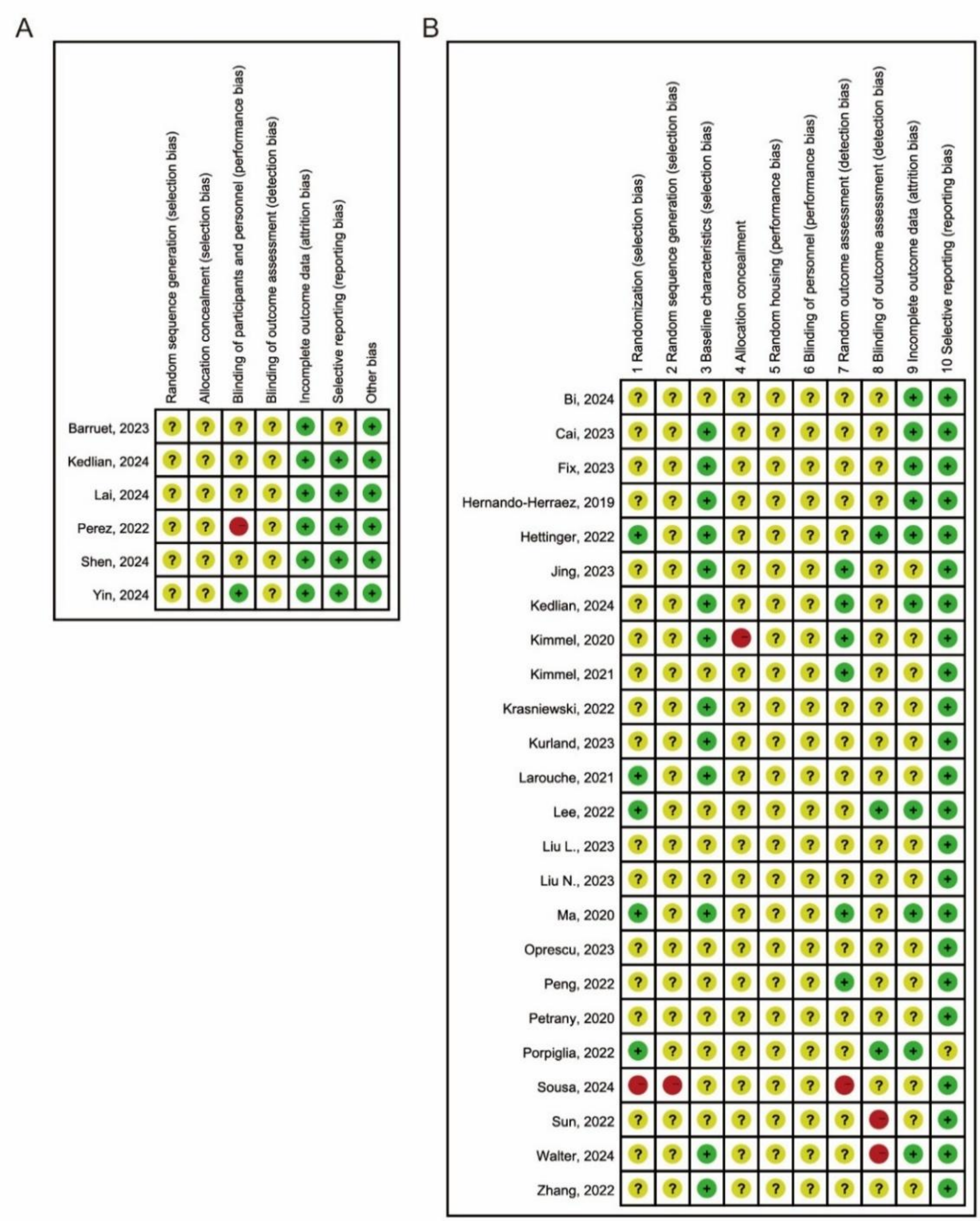

Supplementary Figure 1. Details of risk of bias (RoB). (A) analysis of 6 clinical studies based on Cochrane RoB tool. **(B)** analysis of 24 animal studies analyses based on SYRCLE's RoB tool. Color of symbols: green: low risk; yellow: unclear risk; red: high risk.

© 2025. Liu C et al. Published online at http://www.aginganddisease.org/EN/10.14336/AD.2025.0701

SUPPLEMENTARY DATA

Supplementary Table 1. Sequencing method of included studies

Author	Year	Main model	Sequencing strategy	Sample	Sample preparation	Loaded cell number	System	Libraries
Aging								
Hernando-Herraez et al.	2019	Mice	Single-cell	tibialis anterior (TA)	Fluorescence-activated Cell Sorting (FACS)	~253 (MuSCs) per sample	Smartseq2	Illumina
Kimmel et al.	2020	Mice	Single-cell	TA	FACS	~3636 (MuSCs) per sample	10× Genomics Chromium system	Illumina NovaSeq
Petrany et al.	2020	Mice	Single-nucleus	TA, soleus (SOL)	FACS	~13000 per sample	10× Genomics Chromium system	Illumina NovaSeq 6000
Kimmel et al.	2021	Mice	Single-cell	limb muscle, blood	FACS	10000 per sample	10× Genomics Chromium system	10× Chromium library
Krasniewski et al.	2022	Mice	Single-cell	quadriceps (QUA), gastrocnemius (GAS), TA, SOL, hamstring	FACS	~10000 (macrophages) per sample	10× Genomics Chromium system	Illumina NovaSeq 6000
Peng et al.	2022	Mice	Single-cell	TA	Enzymatic digestion	13612 total (15 samples)	10× Genomics Chromium system	Illumina Novaseq 6000
Perez et al.	2022	Human	Single-nucleus	vastus lateralis (VL)	Singulator instrument (S2 Genomics)	143051 total (17 samples)	10× Genomics Chromium system	Illumina Novaseq 6000
Porpiglia et al.	2022	Mice	Single-cell	TA, GAS	FACS	500 (MuSCs) per sample	Microfluidc RNA-seq chip Fluidigm C1 System	Illumina NextSeq
Zhang et al.	2022	Mice	Single-cell	QUA, GAS, TA, extensor digitorum longus (EDL)	Enzymatic digestion	5000	10× Genomics Chromium system	Illumina HiSeq 4000
Barruet et al.	2023	Human	Single-cell	VL/pectoralis major/rectus abdominis/GAS	FACS	~19482 (MuSCs) per sample	10× Genomics Chromium system	Illumina HiSeq2500, NovaSeq 6000

© 2025. Liu C et al. Published online at http://www.aginganddisease.org/EN/10.14336/AD.2025.0701

Jing et al.	2023	Monkey	Single-nucleus	GAS	FACS	~7000 per sample	10× Genomics Chromium system	Illuminna NovaSeq 6000
Liu N. et al.	2023	Mice	Single-cell (dataset)	TA (GSE132042)	FACS	5000 per sample	GemCode Single-Cell Instrument (10× Genomics)	Illuminna NovaSeq 6000
Kedlian et al.	2024	Human and Mice	Single-cell and single-nucleus	human intercostal muscles and mouse hindlimb muscles	Enzymatic digestion and Percoll gradient centrifugation	8000 per sample	10× Genomics Chromium system	Illumina HiSeq 4000 or NovaSeq 6000
Lai et al.	2024	Human	Single-cell and single-nucleus	semitendinosus, VL and gluteus medius	Enzymatic digestion	~6031 cells and ~9566 nuclei per sample	DNBelab C Series	BGISEQ-2000 or DIPSEQ T1
Shen et al.	2024	Human	Single-nucleus	VL	Singulator instrument (S2 Genomics)	101862 total (17 samples)	10× Genomics Chromium system	Illumina Novaseq 6000
Yin et al.	2024	Human	Single-cell	gluteus maximus and femoral neck	Enzymatic digestion	40238 total (6 samples)	10× Genomics Chromium system	Illumina Novaseq
Bi et al.	2024	Mice	Single-cell (dataset)	TA (GSE143437, GSE162172, GSE232106) QUA, GAS, TA, EDL (GSE172410) QUA, GAS, TA, SOL (GSE195507)	FACS, Enzymatic digestion	34067 total (17 samples)<br>9469 total (macrophages) (6 samples)	10× Genomics Chromium system	Illumina NextSeq 500 (GSE143437, GSE162172, GSE232106) Illumina HiSeq 4000 (GSE172410) Illumina NovaSeq 6000 (GSE195507)
Aging & Regeneration								
Larouche et al.	2021	Mice	Single-cell	TA, EDL, GAS	FACS	6609, ~34930 total (MuSCs)	FACS, 10× Genomics Chromium system	Illumina NextSeq 500 or Illumina NovaSeq

© 2025. Liu C et al. Published online at http://www.aginganddisease.org/EN/10.14336/AD.2025.0701

Cai et al.	2023	Mice	Single-nucleus	QUA	Enzymatic digestion	NA	10× Genomics Chromium platform	Illumina NovaSeq 6000
Sousa et al.	2024	Mice	Single-cell	QUA, TA	FACS	40956 total ($CD45^{+}$ cells) (39 samples)	10× Genomics Chromium platform	Illumina NovaSeq 6001
Regeneration								
Fix et al.	2021	Mice	Single-cell	Soleus muscle	FACS	~ 3700 ($CD45^{+}$ cell) per sample	10× Genomics Chromium system	10× Genomics Cell Ranger pipeline
Lee et al.	2022	Mice	Single-cell	TA	FACS	35836 total (3 samples)	10× Genomics Chromium system	Illumina 2500
Kurland et al.	2023	Mice	Single-nucleus	TA	Mechanical homogenization, sucrose gradient with differential centrifugation	~1800 per sample	Next GEM Single Cell 3' v3.1	Illumina Nextseq500 or Novaseq6000
Oprescu et al.	2023	Mice	Single-cell	TA	FACS	13887 total (6 samples)	10× Genomics Chromium system	IIlumina NovaSeq 6000
Walter et al.	2024	Mice	Single-cell	TA	Enzymatic digestion	273923 total (65 samples)	10× Genomics Chromium system	Illumina NextSeq 500
Aging & Treatment								
Ma et al.	2020	Rats	Single-nucleus	skeletal muscle	FACS	28131 total (6 samples)	10× Genomics Chromium system	Illumina Novaseq 6000
Hettinger et al.	2022	Rats	Single-cell	GAS	FACS	51582 total (6 samples)	10× Genomics Chromium system	Illumina HiSeq platform
Liu L. et al.	2023	Mice	Single-cell	hindlimb muscle	FACS	118449 total (16 samples)	10× Genomics Chromium system	Illumina Novaseq 6000

© 2025. Liu C et al. Published online at http://www.aginganddisease.org/EN/10.14336/AD.2025.0701

Sun et al.	2023	Mice	Single-nucleus	skeletal muscle	FACS	7000 per sample	10× Genomics Chromium system	Illumina Novaseq 6000

Abbreviations: EDL, extensor digitorum longus; FACS, Fluorescence-activated Cell Sorting; GAS, gastrocnemius; MuSCs, muscle stem cells; QUA, quadriceps; SOL, soleus; TA, tibialis anterior; VL, vastus lateralis.

Supplementary Table 2. Study design of included animal studies (aging part)

Author	Year	Main model	Detected cell types
Hernando-Herraez et al.	2019	Male C57BL/6 mice:<br>young: 1.5–2.1 months (n=4)<br>old: 23.2–27 months (n=2)	Satellite cells
Kimmel et al.	2020	Male Histone H2B (H2B)-Green Fluorescent Protein $(GFP)^{+/-}$ reverse tetracycline-controlled transactivator $(rtTA)^{+/-}$ mice:<br>young: 3 months<br>old: 20 months<br>(n=2, per group)	Muscle stem cells (MuSCs)
Petrany et al.	2020	Male C57BL/6 mice:<br>young: 5 months<br>old: 24 months and 30 months<br>(n=1, per group)	Endothelial cells (ECs), smooth muscle, fibro-adipogenic progenitors (FAPs), tenocytes, immune cells, satellite cells, neuromuscular junction (NMJ), myotendinous junction, type IIa myonuclei, type IIx myonuclei, type IIb myonuclei, adipocytes, Schwann cells, undefined
Kimmel et al.	2021	Male C57BL/6J mice:<br>young: 3 months<br>old: 20 months<br>(n=2, per group)	MuSCs, FAPs, muscle mononuclear cells, endothelial cell, T cell, pericyte, skeletal muscle cell, B cell, Schwann cell, tenocyte, macrophage, skeletal muscle myoblast, unknown
Krasniewski et al.	2022	Male and female C57BL/6JN mice:<br>young: 3 months<br>old: 22–24 months<br>(n=3, per group)	Macrophage
Peng et al.	2022	5-month-old SAMP6 mice:<br>sham control P6Sham (n=5)<br><br>Sarcopenic dexamethasone-injected SAMP6 (P6DX) (n=5)<br><br>5-month-old SAMR1 mice:<br>control (n=5)	Fibroblast, glial cells, myocyte, pericyte, neural cells, MuSC, endothelia, macrophage
Porpiglia et al.	2022	Female C57BL/6J mice:<br>young: 2–4 months<br>old: 22–24 months<br>(n=3 per group)	MuSCs
Zhang et al.	2022	Male and female C57BL/6 mice<br>young: 3–5 months<br>old: 20–24 months<br>(n=3 per group)	FAPs, EC, myonuclei, pericytes, macrophages, neurons, satellite cells, tendon cells, neutrophils
Jing et al.	2023	Monkey:<br>young: 4–6 years old<br>old: 18–21 years old<br>(n=3, per group)	Fast IIA, fast IIX, slow I, postsynaptic muscle fibre (PMF), terminal Schwann cell (tSC), muscle stem cell, fibroblast/FAP, tendon fibroblast (TF), smooth muscle cell (SMC), pericytes, endothelial cell, adipocyte, macrophage, T cell

Liu N. et al.	2023	Male C57BL/6J mice:<br>young: 1 and 3 months<br>older: 18 and 21 months<br>oldest: 24 and 30 months<br>(n=3, per group)	Skeletal muscle cell, macrophage, B cell, T cell, mesenchymal stem cell, endothelial cell, skeletal muscle satellite cell, SMC, Schwann cell
Bi et al.	2024	Male and female C57BL6 mice:<br>young: 4–7 months (n =3)<br><br>Female C57BL/6 mice:<br>young: 6 months (n=3)<br>old: 24 months (n=3)<br><br>Female C57BL/6J mice:<br>old: 20 months (n=4)<br><br>C57BL/6J mice:<br>old: 26 months (n=4)<br><br>Male C57BL/6JN mice:<br>young: 3 months (n=3)<br>old: 22–24 months (n=3)	ECs, FAPs, myonuclei, pericytes, smooth muscle cells, macrophages, neurons, tenocytes, satellite cells, neutrophils, B cells
Kedlian et al.	2024	Male C57BL/6JRj mice:<br>young: 3 months (n=5)<br>old: 19 months (n=3)	Adipocyte, neutrophil, other myofiber (MF-other), fast-twitch myofiber (MF-IIB, MF-IIX, MF-IIA), slow-twitch myofiber (MF-I), NMJ, MTJ, tenocyte, fibroblasts (FB), lymph endothelial cells (LymphEC), venous endothelial cells (VenEC), capillary endothelial cells (CapEC), artery endothelial cells (ArtEC), pericyte, red blood cells (RBC), M2_LYVE1+, Mϕ_LAM, monocyte, SMCs, non-myelinating Schwann cells (nmSchwann), myelinating Schwann cells (mSchwann), B cell
Larouche et al.	2021	Female C57Bl/6J WT mice:<br>Young: 4–6 months<br>aged: 19–24 months<br>Female Sod1-/- mice: 10 months<br>Female Sod1-/- rescue (SynTgSod1-/-) mice: 10 months	MuSCs, mesenchymal stem cells (MSC), tenocyte, CD45+ immune cell, NMJ, endothelial cell, SMC
Cai et al.	2023	Male C57BL/6J mice:<br>young: 2-month-old<br>old: 22-month-old	Myotendinous junction (MTJ), tenocytes, fast-IIA, fast-IIX, fast-IIB, myoblast, FAP, MuSC, SMC, ECs (VenEC, CapEC, ArtEC), cytotoxic T cell, dendritic cells (DC), pericytes, adipocytes, immune cells (Monocytes, *Arg1*+ Mac, macrophage-1 (Mac 1) and macrophage-2 (Mac 2))
Sousa et al.	2024	Female C57BL/6 mice:<br>young: 3 months<br>old: 25 months	Monocyte/Macrophage, granulocyte (eosinophils, neutrophils), fibrocytes, DC-1, DC-2, DC-3, lymphocyte (B cells 1, B cells 2, T cells, type 2 innate lymphoid cells (ILC2s) and NKs)
Ma et al.	2020	Male and female Wild-type Sprague-Dawley (SD) rats:<br>young: 5 months<br>old: 27 months<br>(n=6 per group)	NMJ, TF, SMC, perivascular macrophage, satellite cells, type I, Pericytes, EC, stromal cell (ST), type IIA, type IIX
Hettinger et al.	2022	Male Fisher 344 X Brown Norway (F344/BN) rats:<br>adult: 10 months<br>aged: 30 months<br>(n=1, per group)	Pericytes, EC, tenocytes, FAPs, monocytes, T cells, NK cells, mast cells, satellite cells, macrophages, antigen presenting cells, granulocytes, SMCs
Liu et al.	2023	Male C57BL/6J mice:<br>control young mice (Y-C): 4 months<br>control old mice (O-C): 22 months<br>(n=4, per group)	B cells, T cells, monocytes, macrophages, Schwann cells, SMCs, ECs, FAPs, tenocytes, MuSCs

© 2025. Liu C et al. Published online at http://www.aginganddisease.org/EN/10.14336/AD.2025.0701

Sun et al.	2023	Male C57BL/6J mice:<br>young control (Y-Ctrl): 2 months<br>old control (O-Ctrl): 16 months<br>(n=22–24, per group)	Pericyte, FAP, SMC, EC, tendon, satellite cell, fast-IIA, fast-IIX, fast-IIB, slow-I, NMJ-pre, NMJ-post, adipocyte, T cell, macrophage, myoblast, NMJ

Abbreviations: ArtEC, artery endothelial cells; CapEC, capillary endothelial cells; cDC1, Conventional type 1 dendritic cell; cDC2, Conventional type 2 dendritic cell; DC, dendritic cells; ECs, Endothelial cells; EnFB, endoneurial fibroblasts; FAPs, Fibro-adipogenic progenitors; GFP, Green Fluorescent Protein; H2B, Histone H2B; ILC2s, type 2 innate lymphoid cells; LymphEC, lymph endothelial cells; Mac, macrophage; MF-I, slow-twitch myofiber; MF-II, fast-twitch myofiber; MTJ, Myotendinous junction; MSC, mesenchymal stem cells; mSchwann, myelinating Schwann cells; MuSCs, Muscle stem cells; NMJ, neuromuscular junction; NK cell, natural killer cell; nmSchwann, non-myelinating Schwann cells; PMF, postsynaptic muscle fibre; PnFB, perineural fibroblasts; rtTA, Reverse tetracycline-controlled transactivator; SMC, Smooth muscle cell; ST, stromal cell; TF, tendon fibroblast; tSC, terminal Schwann cell; VenEC, venous endothelial cells.

Supplementary Table 3. Study design of included human studies (aging part)

Author	Year	Main model	Detected cell types
Perez et al.	2022	Human (Canada):<br>young: 18 – 30 years old (n=6)<br>old: 65 – 85 years old (n=11)	Type II fibers, type I fibers, Fibro-adipogenic progenitors (FAPs), satellite cells, smooth muscle cells (SMC), endothelial cells (EC), immune cells (macrophages, T/Natural Killer (NK) cells)
Barruet et al.	2023	Human (USA):<br>young: <30 years old (n =2)<br>adult: 35 – 66 years old (n=7)<br>aged: >70 years old (n=3)	Muscle stem cells (MuSCs), muscle progenitors, myoblasts, myocytes, mesenchymal cells
Kedlian et al.	2024	Human (UK and China):<br>young: 20 – 40 years old (n=8)<br>old: 60 – 75 years old (n=9)	SMC, pericyte, lymph endothelial cells (LymphEC), venous endothelial cells (VenEC), capillary endothelial cells (CapEC), artery endothelial cells (ArtEC), mast cells, Conventional type 2 dendritic cell (cDC2), Conventional type 1 dendritic cell (cDC1), macrophage, monocyte, B-plasma, B cell, natural killer cells (NK cell), T cell, adipocyte, non-myelinating Schwann cells (nmSchwann), myelinating Schwann cells (mSchwann), tenocyte, perineural fibroblasts (PnFB), endoneurial fibroblasts (EnFB), fibroblasts (FB), MuSC, MF-IIsc (fg), fast-twitch myofiber (MF-II), MF-Isc (fg), slow-twitch myofiber (MF-I)
Lai et al.	2024	Human (Spain and China):<br>adults: 15 – 46 years old (n=12)<br>older adults: 74 – 99 years old (n=19)	MuSCs, FAPs, fibroblast-like cells and adipocytes, pericytes, SMC, ECs, myeloid, lymphocyte, mast cells, Schwann cells, specialized myofibers, type I, type II
Shen et al.	2024	Human (USA):<br>young: n=6<br>old: n=11	Type IIa, type I, mixed type I/II, FAPs, ECs, type II_2, pericytes, immune cells (M2 macrophage, T cell, NK cell, M1 macrophage, mast cell and DC)
Yin et al.	2024	Human (China):<br>young: 29 and 34 years old (n=2)<br>old: aged >70 years (n=4)	ECs, fibroblasts (FBs), lymphatic ECs, mesenchymal stem cells (MSCs), pericytes, SMCs, myocytes, satellite cells, myeloid cells, lymphoid cells, plasma B cells, mast cells, cycling cells

Abbreviations: ArtEC, artery endothelial cells; CapEC, capillary endothelial cells; cDC1, Conventional type 1 dendritic cell; cDC2, Conventional type 2 dendritic cell; DC, dendritic cells; ECs, Endothelial cells; EnFB, endoneurial fibroblasts; FAPs, Fibro-adipogenic progenitors; LAMs, lipid-associated macrophages; LymphEC, lymph endothelial cells; Mac, macrophage; MF-I, slow-twitch myofiber; MF-II, fast-twitch myofiber; MSC, mesenchymal stem cells; mSchwann, myelinating Schwann cells; MuSCs, Muscle stem cells; NK cell, natural killer cells; nmSchwann, non-myelinating Schwann cells; PnFB, perineural fibroblasts; SMC, Smooth muscle cells; VenEC, venous endothelial cells.

© 2025. Liu C et al. Published online at http://www.aginganddisease.org/EN/10.14336/AD.2025.0701

SUPPLEMENTARY DATA

Supplementary Table 4. Study design of included animal studies (regeneration & treatment part)

Author	Year	Model	Intervention / Treatment	Detected cell types
Regeneration				
Larouche et al.	2021	Female C57BL/6J WT mice:<br>young: 2–3 months<br>aged: 22–24 months<br><br>Female *Sod1*$^{-/-}$ mice: 10–12 months<br><br>Female *Sod1*$^{-/-}$ rescue (*SynTgSod1*$^{-/-}$) mice: 10–12 months	Injury of hind limb muscles: intramuscular $BaCl_2$ injection in old mice<br><br>Sciatic nerve transection (SNT) to *Sod1*$^{-/-}$ mice	Muscle stem cells (MuSCs), mesenchymal stromal cell (MSC), tenocyte, immune cell, neuromuscular junction, (NMJ), endothelial cells (ECs), smooth muscle cells (SMC)
Cai et al.	2023	Male C57BL/6J male mice:<br>young: 2-month-old<br>old: 22-month-old	Cryoinjury of the quadriceps femoris: pre-chilled metal rod with liquid nitrogen was applied to the exposed quadriceps femoris for 10 sec (n=4–13, per group)	Myotendinous junction (MTJ), tenocytes, Fast-IIA, Fast-IIX, Fast-IIB, myoblast, FAP, MuSC, SMC, venous endothelial cells (VenEC), capillary endothelial cells (CapEC), artery endothelial cells (ArtEC), cytotoxic T cell, dendritic cells (DC), Pericytes, adipocytes, (Monocytes, *Arg1*+ Mac, Mac 1 and Mac 2)
Fix et al.	2021	Male C57BL/6 mice:<br>young: 4–5 months<br>old: 20–22 months	1. Ambulatory controls (CON) (young: n=10, old: n=20)<br>2. 14 days of hindlimb unloading (HU) (young: n=8, old: n=10)<br>3. HU followed by 4 days of reloading (RL4) (young and old: each n=10)	CD45+ cells: *IL-1β*+, *CCL8*+, *Spp1*+ and *S100a9*+ macrophages, neutrophils, *Ly6C*+ and *Ly6C*- monocytes, *Cd209*+ dendritic cells, innate lymphoid cells, CD8 memory T cells, *IRF8*+ dendritic cells, Retnl-alpha dendritic cells, gamma delta T cells, endothelial progenitor cells (EPC), CD8 central memory T cells, B cells, natural killer cells, immature T cells, ST, lymph node monocyte cells, fibrocytes, CD8 effector T cells, mast cells, basophils
Lee et al.	2022	Male C57BL/6J mice:<br>young: 3–4 months<br>old: 24–26 months<br>(Number of mice is not mentioned)	Injury of tibialis anterior muscles: $BaCl_2$ injection treated with three treatments of<br>1. Control: Phosphate buffered saline (PBS) (young and old)<br>2. Treatment: 0.1 ug<br>3. Recombinant meteorin-like (rMETRNL) (old) after 24h and 48h BaCl2 administration	Immune cells, neuron, Fibro-adipogenic progenitors (FAPs), T cells, fibroblast/tenocytes, MuSC/myoblast, myocytes, pericytes, endothelial cells, erythrocytes
Kurland et al.	2023	Young male and female C57B6 mice: 4–8 months<br>Aged male and female F1 mice from a C57BL/6J and DBA/2J cross: 24–28 months<br><br>For SnRNA seq: on TA muscles of young and aged mice at<br>0 dpi: uninjured (n=2, per group)<br>4 dpi: myonuclear production is largely complete (young=2, aged=3)	Injury of Tibialis anterior (TA) and extensor digitorum longus (EDL) muscle: Injection with BaCl2, injured young and aged mice were then treated with ethynyl 20-deoxyuridine (EdU) for 6 h before tissue harvesting	Immune cells, endothelial cells, MuSCs, progenitors, myonuclei, fibroblast, smooth muscle, unidentified

		7 dpi: myonuclei are likely maturing (n=2, per group)		
Oprescu et al.	2023	Male *Pax7nGFP/+* mice at 7 days post injury (DPI):<br>young: 3–4 months<br>old: 20 months<br>(n=3, per group)	Injury: Intramuscular injection of 50-µL 10-µM cardiotoxin	MuSCs, FAPs, SMCs, ECs, macrophage (3 populations)
Sousa et al.	2024	Female C57BL/6 mice:<br>young: 3 months<br>old: 25 months	Injury: intramuscular injection of 50 µl or 40 µl 1.2% barium chloride in sterile saline solution into the quadriceps muscles or TA respectively	Monocyte/Macrophage, granulocyte (eosinophils, neutrophils), fibrocytes, dendritic cells (DC-1, DC-2, DC-3), lymphocyte: B cells 1, B cells 2, T cells, type 2 innate lymphoid cells (ILC2s) and NKs
Walter et al.	2024	Male and female C57BL/6J mice:<br>young: 4–7 months<br>old: 20 months<br>geriatric: 26 months<br>(n=3–4, per age and dpi)	Injury of TA muscles: injection of 10 µl Notexin	Monocyte/macrophage, (cycling, *Cxcl10+*, patrolling, *Ccr2+*, *Mrc1+* and *Cx3cr1+*), dendritic cells (*Fscn1+, Xcr1+, Cd72+* and *Cd209a+*), neutrophils, T cells (non-cycling, cycling and Cd4+), NK cells, B cells, erythrocytes, FAPs (stem, pro-remodeling and adipogenic), tenocytes, Schwann and neural/glial cells, MuSCs and progenitors, myonuclei, pericytes and smooth muscle cells, endothelial cells (artery, capillary and vein), endothelial, myeloid cells
Treatment				
Ma et al.	2020	Male and female Wild-type Sprague-Dawley (SD) rats:<br>young: 5 months<br>old: 27 months<br>(n=6, per group)	1.Y-ad libitum (Y-AL) (n=6)<br>2. O-AL (n=6)<br>3. O-CR (n=6)<br>9-month caloric restriction (CR): food intake of rats ↓ 10% per week to a final 70% of AL food intake	NMJ, tendon fibroblast (TF), SMC, perivascular macrophage, satellite cells, type I, pericytes, EC, stromal cell, type IIA, type IIX
Hettinger et al.	2022	Male Fisher 344 X Brown Norway (F344/BN) rats:<br>adult: 10 months<br>aged: 30 months<br><br>For scRNA seq: (n=6)<br>(n=1, per group)	1. Weight bearing (WB) for 21 days<br>2. Recovery after atrophy without mechanotherapy (RE): hindlimb suspension for 14 days and reloading for 7 days<br>3. Recovery after atrophy with mechanotherapy (REM): hindlimb suspension for 14 days, reloading and mechanotherapy for 7 days (mechanotherapy: four 30-minute session for 1 week)	Pericytes, endothelial cells, tenocytes, FAPs, monocytes, T cells, natural killer cells, mast cells, satellite cells, macrophages, antigen presenting cells, granulocytes, smooth muscle cells
Liu et al.	2023	Male C57BL/6J mice:<br>young: 4 months<br>old: 22 months<br>1. Control young mice (Y-C)<br>2. Exercised young mice (Y-Ex)<br>3. Control old mice (O-C)<br>4. Exercised old mice (O-Ex)<br>(n=4, per group)	Treatment: mice were housed individually for 5 weeks for voluntary wheel running<br><br>Control: mice were singly housed without the wheels	B cells, T cells, monocytes, macrophages, Schwann, SMCs, ECs, FAPs, tenocytes, MuSCs
Sun et al.	2023	Male C57BL/6J mice:<br>young: 2 months<br>old: 16 months	Treatment: access to wheels for 12 months in activity cages	Pericyte, FAP, SMC, EC, tendon, satellite cell, fast_IIA, fast_IIX, fast_IIB, slow_I, NMJ_pre,

		1. Young control (Y-Ctrl)<br>2. Young exercise (Y-Ex)<br>3. Old control (O-Ctrl)<br>4. Old exercise (O-Ex)<br>(n=22–24, per group)	Control: access to standard animal facility cages	NMJ_post, adipocyte, T cell, macrophage, myoblast, NMJ

Abbreviations: ArtEC, artery endothelial cells; CapEC, capillary endothelial cells; CR, caloric restriction; DC, dendritic cells; ECs, Endothelial cells; EPC, endothelial progenitor cells, FACS, Fluorescence-activated cell sorting; FAPs, Fibro-adipogenic progenitors; FSMs, Fluorescence-activated cell sorting-sorted MuSCs; ILC2s, type 2 innate lymphoid cells; Mac, macrophage, MF-I, slow-twitch myofiber; MF-II, fast-twitch myofiber; MTJ, Myotendinous junction; ; MuSCs, Muscle stem cells; Myo, myoblast; NK cell, natural killer cells; NMJ, neuromuscular junction, NMJ_post, post- neuromuscular junction; NMJ_pre, pre-neuromuscular junction; rMETRNL, Recombinant meteorin-like; SMC, Smooth muscle cells; ST, stromal cell; SNT, Sciatic nerve transection; TF, tendon fibroblast; VenEC, venous endothelial cells.

Supplementary Table 5. Marker genes of muscle cell types

	Cell Type	Marker gene
Muscle Cell	Myocyte	MYOD1, MYOG, MYH2, CDKN1C, SOD2, GNAS, JUNB, FOS (Barruet)<br>Myh11, Acta2, Smtn (Peng)<br>MYL2, CKM, ACTA1, TNNC2, MB (Yin)<br>Dclk1, Gm28653, Dlg2, Dpp6, Ncam1, Sntb1, Myh3, Gse1, Prune2, Pkhd1 (Cai)<br>Ptprc, Cd52, Acta1, Col3a1 (Lee)<br>**RASA4+:** FLCN, FNIP1, RASA4<br>**MYH8+:** TNNT3, CKM, MB, SNX7, CDKN1A, SAA1, MYH8 (Kedlian)
	Type I fiber	**Slow I:** MYH7, TNNT1, TNNI1 (Jing)<br>TNNT1, MYH7/7B, ATP2A2, LGR5, XPO4 (Perez)<br>MYH7, TNNT1, TNNI1, MYH7B (Lai)<br>Myh7 (Ma)<br>TNNC1, TNNI1, TNNT1, TPM3, ATP2A2, MYH7, MYL2, MYL3;<br>**Mixed Type I/II:** TNNC1, TNNI1, TNNT1, TPM3, ATP2A2, MYH7, MYL2, MYL3, ATP2A1, ENO3, MYBPC2, MYH2, PEKM, TNNT3, TPM1, MYL12A, GADPH, MYLPF, TNNC2, TNNI2 (Shen)<br>**MF-I:** MYH7, TNNT1<br>**MF-Isn (fg):** MYH7, TNNT1, CKM, MB<br>**MF-Isc (fg):** MYH7, TNNT1, CKM, MB (Kedlian)<br>**Slow I:** Myh7, Ankrd2, Gm28653, Myom3, Gm31251, Myl2, Csrp3, Myoz2, Myh7b, Tnnt1, Myh10, Tnnc1, Mhrt, Fhl1, Lmod2, Atp2a2, Myh2, Lmcd1, Cryab, Actn2, Mybpc1, Atf3, Tpm3, Flnc, Dkk2, Enah, Ankrd1, Klhl40, Gm13601, Xirp2 (Sun)
	Type II fiber	MYH1, MYH2, TNNT3, TNNI2 (Lai)<br>TNNT3, MYH1/2<br>**Fast skeletal MC_1:** MYH1, TNNC2, MYLPF, TNNI2, ACTN3<br>**Fast skeletal MC_2:** TNNT3, IGFN1, ATP2A1, MYLK4, COL4A3<br>**Fast skeletal MC_3:** APOD, DCN, HBB (Perez)<br>**IIA:** MYH2, MYH1, TNNT3 (Jing)<br>**IIX:** MYH2, MYH1 (Jing)<br>**Type IIB:** Myh4, Ttn, Pde4d, Mylk4, Tpm1, Sorbs, Phka1, Actn3, Atp2a1, Trdn, Ampd1<br>**Type IIX:** Myh1, Ttn, Agbl1, 4831440D22Rik, Ppara, Sorbs1, Ptpn3, Cd36, Pank1, Prune2<br>**Type IIA:** Myh2+ (Petrany)<br>**MF-II:** TNNT3<br>**MF-IIsn (fg):** TNNT3, MYH1, MYH2, CKM, MB<br>**MF-IIsc (fg):** TNNT3, MYH1, MYH2, CKM, MB (Kedlian)<br>**IIA:** ATP2A1, ENO3, MYBPC2, MYH2, PEKM, TNNT3, TPM1 (Shen)<br>**Fast IIB:** Myh4, Pfkfb3, Actn3, Mylk4, Pde4d, Mybpc2, Pvalb, Eya4, Ampd1, Phka1, Pgm1, Trim63, Sorbs2, Clip1, Rbfox1, Prkag3, Mylk2, Xpr1, Ighm, Fbxo32, Ttll7, Tpm1, Ube2d1, Tmem233, Casq1, Gm26740, Sox6, Phkg1, Agl, Trdn<br>**Fast IIX:** Myh1, Agbl1, Mybpc1, Esrrg, Ank2, Actn2, Cd36, Ckmt2, 2310016D03Rik, Vegfa, Ptpn3, Fhl1, Atp1b1, Padi2, Adk, 4831440D22Rik, Kcnn2, Ppara, Wnt5b, Filip1l, Ppargc1a, Tmem65, 2310067P03Rik, Rmdn1, Fgf1, Sorbs1, Coq8a, Ppargc1b, Qk, Acss2<br>**Fast IIA:** Myh2, Ppara, Agbl1, Esrrg, Ankrd2, Myom3, Actn2, Ckmt2, Myoz2, Lpl, Lmod2, Mybpc1, Grm1, Sox2ot, Coro6, Idh2, Qk, Atp1b1, Lmcd1, Ank2, Sorbs1, Ptpn3, Fhl1, Gm41757,

© 2025. Liu C et al. Published online at http://www.aginganddisease.org/EN/10.14336/AD.2025.0701

		Arpp21, Gm28653, Acss2, Fgf1, Ldhb, Adk (Sun)<br>**Fast IIA:** Myh2, Agbl1, Esrrg, Myh1, Actn2, Ppara, Mybpc1, Ckmt2, Ankrd2, Fhl1<br>**Fast IIB:** Myh4, Mylk4, Phka1, Trdn, Atp2a1, Phkg1, Rbfox1, Ctnna3, Mybpc2, Ckm<br>**Fast IIX:** Agbl1, Myh1, Esrrg, Mybpc1, Ckmt2, Actn2, 2310016D03Rik, Vegfa, Kcnn2, Ank2 (Cai)<br>**IIA:** Myh1, Ncam1, Musk<br>**IIX:** Myh1 (Ma)
	Myonuclei	Acta1, Mylpf, Tnnt3, Tnnc2, Myl1, Tnni2, Ckm, Tpm1, Emo3, Pvalb (Zhang)<br>Neb (Kurland)<br>Acta1, Tnnt3, Tnnc2, Ckm, Mylpf (Larouche)<br>Acta1, Mylpf, Tnnt3, Tnnc2, Myl1 (Bi)<br>Acta1, Myh1, Myh4 (Walter)<br>Nr4a3+ myonuclei: Hk2, Nr4a3, Nos1, Optn, Fam134b, Trim63, Ppargc1a, Map3k14, Ankrd33b, Prkag2<br>Enah+ myonuclei: Atf3, Flnc, Lmcd1, Xirp2, Fhl1, Pdlim3, Enah<br>Ampd3+ myonuclei: 5730419F03Rik, Gm47782, Dupd1, Dapk2, Kcnq5, Pvt1, Runx1, Mdm4, Malat1, Gm28653 (Petrany)
	Neuromuscular junction (NMJ)	Chrne, Etv5, Colq, Musk, Pdzrn4, Hs6st2, Col19a1, Ano4, Ncam1, Phldb2, Gramd1b, Prkar1a, Vav3, Ablim2 (Petrany)<br>MUSK, ACHE (Kedlian)<br>Ncam1, Musk (Ma)<br>Gpm6b, Mpz, Lyz2, Plp1, Ptn (Larouche)<br>**NMJ_post:** Ano4, Prkar1a, Phldb2, Ncam1, Colq, Col19a1, Ablim2, Pdzrn4, Hs6st2, Gramd1b, Chrne, Col4a3, Vav3, Lrfn5, Rps6ka1, Etv5, Calcrl, Map3k7cl, Ryr3, Ufsp1, Epb41l4a, Col13a1, B4galnt3, Utrn, Cfap54, Akap11, Ache, Musk, Ank3, Kcnq5<br>**NMJ_pre:** Cdh19, Kank4, Mpz, Lgi4, Csmd1, Matn2, Gm2164, Gm10800, Grik2, Gm10801, Slc35f1, Adgrl3, Nkain2, Reln, Gm20754, Negr1, Hcn1, Chl1, Il1rapl1, Cadm2, Lrrc4c, Lsamp, Pcdh9, Frmd4a, Mbp, Csmd3, Sorcs1, Trpm3, Stard13, Glis3 (Sun)
	Myotendinous junction	Col22a1, Ankrd1, Slc24a2, Adamts20, Fras1, Ldlrad3, Lama2, Atrnl1, Nav3, Erbb4 (Petrany)<br>MYH7, TNNT1, TNNT3, CKM, MB (Kedlian)<br>Col22a1, Erbb4, Lama2, Slc24a2, Atp13a5, Col24a1, Limch1, Fras1, Adamts20, Prkg1, App, Sgcd, Rgcc, Xirp2, Gm34804, Atp1b4, Net1, Dennd5b, Usp6nl, Ldlrad3<br>Sorbs2, Med12l, Atrnl1, Mss51, Cobl, Hivep3, Kcnh7, Angpt, Nav, Frmpd4 (Sun)<br>Ano4, Ablim2, Prkar1a, Pdzrn4, Col4a3, Col19a1, Colq, Phldb2, Tafa4, Kcnq5 (Cai)
	Myoblast	SOX8, MYOD1, MYOG, CDKN1C, SPARC, SOD2, GNAS, JUNB, FOS (Barruet)<br>Myog (Kimmel2)<br>Myh3, Tnnt2, Myh8, Gm28653, Gm11149, Acta2, Cers6, Ncam1, Megf10, Sytl2. Slc9a9, Col19a1, Dclk1, Sntb1, Ccdc141, Ryr3, Map1b, Runx1, Pgm5, Arpp21, Dlg2, Enah, Erf, Sorcs2, Macf1, Nav2, Hip1, Frmd4b, Fggy, Col23a1 (Sun)
	Muscle stem cell (MuSC)	Pax7, Asb5, Myf5, Chodl, Meg3, Peg3, Sdc4, Vcam1, Dag1, Crll1, Nppc (Zhang)<br>NCAM1, MUSK, CADM2, PAX7, CALCR (Jing)<br>Pax7 (Kimmel2)<br>Myod1, Myf5, Gal, Asb5 (Peng)<br>Pax7, HMCN2, MEG3, KCNQ1OT1, CLCN5 (Perez)<br>Pax7, Hs6st3, Fgfr4, Tenm4, CHodl, Kirrel3, Thsd4, Bmp6, Ror1, Gnas (Petrany)<br>APOC1, PAX7, MYF5, DLK1, NNMT (Yin)<br>PAX7, DLK1 (Kedlian)<br>PAX7, MYF5, DLK1 (Lai)<br>APOC1, APOE, MYF5 (Shen)<br>Pax7, Myf5 (Liu L.)<br>Hs6st3, Chodl, Pax7, Csmd1, Ror1, Tenm4, Sema6a, Kirrel3, Col19a1, Peg3, Fgfr4, Tanc2, Cfh, Calcr, Pde1c, Meg3, Sytl2, Gm26834, Hmcn2, Megf10, Ncam1, Clmn, Hs6st2, Sulf1, Tshz3, Pde10a, Cdk14, Slc7a11, Commd10, Nkain2 (Sun)<br>Pax7, Hmga2, Pde1c, Npas3, Csmd1, Notch3, Ptprd, Tenm4, Thsd4, Adgrl3 (Cai)<br>Pax7 (Ma)<br>PAX7 (Hettinger)<br>Pax7, Col3a1 (Lee)<br>Neb, Pax7 (Kurland)<br>Pax7, Postn, Top2a (Oprescu)<br>Pax7, myod1, Pecam1, Myf5, Asb5, Nppc, Chodl, Crl11 (Larouche)<br>Chodl , Peg3 , Sdc4 , Crlf1 ,Pax7, Nppc (Bi)<br>Pax7, Sdc1 (Walter)

© 2025. Liu C et al. Published online at http://www.aginganddisease.org/EN/10.14336/AD.2025.0701

		Hu-MuSCs 1: PAX7, CDKN1C, SPRY1, SPARC, CXCL14, GNAS, JUNB, FOS<br>Hu-MuSCs 2: PAX7, CDKN1C, SPRY1, SPARC, SOD2, CXCL14, GNAS, JUNB, FOS<br>Hu-MuSCs 3: PAX7, CDKN1C, SPRY1, COL1A1, SPARC, CXCL14, GNAS, JUNB, FOS<br>Hu-MuSCs 4: PAX7, CDKN1C, SPRY1, SPARC, SOD2, CXCL14, GNAS, JUNB, FOS<br>Hu-MuSCs 5: PAX7, CDKN1C, SPRY1, COL1A1, SPARC, CXCL14, GNAS, JUNB, FOS<br>Hu-MuSCs 6: PAX7, CDKN1C, SPRY1, IGFBP7, COL1A1, SPARC, CCL2, CXCL14, GNAS, JUNB, FOS<br>Hu-MuSCs 7: PAX7, CDKN1C, SPRY1, SPARC, CXCL14, GNAS, JUNB, FOS<br>Hu-MuSCs 8: PAX7, CDKN1C, HEY1, SPRY1, COL1A1, SPARC, SOD2, MX1, ISG15, CXCL14, GNAS, JUNB, FOS<br>Cycling MuSCs: PAX7, SOX8, MYOD1, TOP2A, PCNA, CDKN1C, SPRY1, COL1A1, SPARC, SOD2, CXCL14, GNAS, JUNB, FOS (Barruet)<br><br>Subpopulation:<br>Cluster 1: Myf5, Pax7, Cd34, Vcam1, Itga7, Spry1<br>Cluster 2: Myf5, Cd34, Vcam1, Spry1<br>Cluster 3: Myod1, Itga7, Esd, Bax, Phlda3, Rps27l, Phgdh, Psma7, Eif3c, Nme1<br>Cluster 4: Myod1, Esd, Bax, Phlda3, Rps27l, Phgdh, Psma7, Eif3c, Nme1, Ythdc1 (Kimmel)<br>Cluster 0: Cxcl1, Tnfaip6, Gbp2, Cluster 1: Sparc, Hk2, Eif4ebp1, Cluster 2: Hspa1a, Hspa1b, Hsbp1, Cluster 3: mt-Co3, mt-Nd2, mt-Cytb (Liu. L)
	Muscle progenitor cells	PAX7, SOX8, CDKN1C, SPRY1, SPARC, CXCL14, GNAS, JUNB, FOS (Barruet)<br>Neb, Myog (Kurland)
	Fibro-adipogenic progenitor (FAP)	Dcn, Ly6a, Pdgfra, Gsn, Smoc2, Myoc, Col3a1, Lum, Clec3b, Apod, Ccl11 (Zhang)<br>FBN1, DCN, GSN (Jing)<br>Pdgfrα, Plin1 (Kimmel2)<br>Pdgfrα, TNXB, ABCA8, CFD, COL6A1, HMCN2 (Perez)<br>Dcn, Hmcn2, Arhgap24, Abca8a, Plxdc2, Egfr, Slit3, Tnxb, Lvrn, Bicc1, Fbn1, Ccnd3, Dlc1, Lama2, Ror1, Itgbl1, Nox4, Eya2, Abi3bp (Petrany)<br>PTGDS, SCN7A, MYOC (Lai)<br>Abca8a, Plxdc2, Lvrn, Egfr, Dcn, Arhgap24, Tnxb, Abca6, Hmcn2, Slit3, Abca8b, Nova1, Bmper, C7, Fbn1, Bicc1, Celf2, Gsn, Scara5, Ebf2, Pdgfra, Naaladl2, Abca9, Thsd7a, Man1a, Dpep1, Antxr2, Nav1, Fap (Sun)<br>Slit3, Meg3, Col3a1, Ebf1, Postn, Egfr, Plxdc2, Ccbe1, Col5a2, Adam12 (Cai)<br>PDGFRA (Hettinger)<br>Pdgfra, Col3a1 (Lee)<br>Postn (Oprescu)<br>Cxcl14, Smoc2, Lum, Apod, Gsn, Dcn, Ccl11, Pi16, Fn1, Fbn1, Igfbp5, Mfap5 (Bi)<br>Stem FAPs: Pdgfra, Col3a1, Igfbp5, Dpp4, Cd34, Gsn, Mmp2<br>pro-remodeling FAPs: Pdgfra, Col3a1, Cdk1, Tyms, Tnfaip6, Il33, Adam12, Bgn, Hdlbp<br>adipogenic FAPs: Pdgfra, Col3a1, Adam12, Bmp5, Myoc, Col1a1, Dcn, Mmp2, Apod (Walter)<br>FBN1+ FAPs: DCN, PDGFRA, FBN1, PRG4, PCOLCE2<br>LUM FAPs: APOD, LUM, DCN, PDGFRA, FBN1 (Shen)<br>Pdgfra (Liu L.)<br><br>Subpopulation:<br>Cluster 3 (p16high): Spp1, Cdkn2a, Ier5, Cxcl1, Ccl2, Ccl7, Thbs1, Cpxm1, Osr2, Apoe, Gpc3, Vwa1, Matn2, Sec11c, Apod, Cp, Thbs4, Angptl7, Rgs16, Rasgrp2, Tenm2 (Zhang)
	Mesenchymal stem cells	PAX7, CDKN1C, SPRY1, IGFBP7, COL1A1, CAV1, SPARC, SOD2, CCL2, GNAS, JUNB, FOS (Barruet)<br>COL1A1, COL3A1, COL1A2, POSTN, FN1, LEPR (Yin)<br>**Stomal cell:** Dcn (Ma)<br>Pdgfra, Gsn, Dcn, Cxcl14, Igfbp6, Ptx3 (Larouche)
Microenvironment	Endothelial cell	Fabp4, Pecam1, Cdh5, Ly6a, Aqb1, Flt1, Gpihbp1, Timp4, Cd36, Adgrf5, Egfl7 (Zhang)<br>Myonuclei: Kdr, Tnnc2, Acta1, Mylpf, Tnnt3, Myl1, Tnni2, Ckm, Tpm1, Eno3, Pvalb (Zhang)<br>PECAM1, FLT1, VWF (Jing)<br>Cdh5 (Kimmel2)<br>PECAM1, FLT1, CDH5, CD34, VWF, JAM2 (Lai)<br>Pecam1 (Liu L.)<br>Flt1, Mecom, St6galnac3, Cyyr1, Adgrl4, Ablim1, Adgrf5, Dach1, Ablim3, Ptprb, Plcb4, Fli1, Prkch, Shank3, Itpkb, Bmp6, Pecam1, Egfl7, Pkp4, Magi1, Mcf2l, Pdgfd, Plcb1, Rasgrf2, Myo10, Mcc, Kitl, Sema6a, Lnx1, Ptprm (Sun)

© 2025. Liu C et al. Published online at http://www.aginganddisease.org/EN/10.14336/AD.2025.0701

		Flt1 (Ma)<br>FLT1 (Hettinger)<br>Ptprc, Cd52, Pecam1, Col3a1 (Lee)<br>Pecam1 (Kurland)<br>Prcam1 (Oprescu)<br>Pecam1, Aqp1, Fabp4, Cldn5, Rnd1, Ccl21a (Larouche)<br>Aqp1, Gpihbp1, Fabp4, Timp4, Egfl7, Tspan13, Cdh5, Stmn2, Tm4sf1, Rbp7, Bsg, Sema3g, Plvap, Lrg1, Selp, Rasa4, Spint2, Tmem252 (Bi)<br>PECAM1, CD34, BTNL9, EFGL7, VWF, PTPRB, SLC9A3R2 (Perez)<br>Pecam1, Shank3, Prkch, Flt1, St6gainac3, Cyyr1, Adgrl4, Ptprb, Mecom, Mecom, Plcb4 (Petrany)<br>TM4SF1, VWF, CLDN5, FABP4, CXCL2 (Yin)<br>Endothelial1: Pecam1, Cldn5, Cd34, Timp4, Aqp1, Gpihbp1<br>Endothelial2: Gbp2, Gbp4 Gbp7, Ifit3, Ifi47, Ifit1<br>Endothelial3: Clu, Stmn2, Bsg, Mgp<br>Endothelial4: Plvap, Selp, Lrg1, Ackr1, Vwf (Peng)<br>lymphatic EC: CCL21, TFF3, TFF2, AGR2, SPINK1 (Yin)<br>Mesothelium: MSLN, ITLN1 (Kedlian)<br>ArtEC: PECAM1, HEY1<br>CapEC: PECAM1, CA4, LPL<br>VenEC: PECAM1, CLU, ACKR1, SELE, RASA4, CDKN1A<br>LymphEC: LYVE1, TFF3 (Kedlian)<br>AEC: Pdgfd, Mecom, St6galnac3, Flt1, Cyyr1, Ptprb, Ablim1, Dach1, Prex2, Pecam1<br>CEC: Flt1, Mecom, Adgrl4, Adgrf5, Cyyr1, Mcf21, Ablim1, Dach1, Ccdc85a, Plcb4<br>VEC: Mecom, Stox2, Tll1, Ptprb, Pecam1, Dnm3, Ldb2, Magi1, Igfbp7, Shank3 (Cai)<br>artery EC: Cdh5, Pecam1, Alpl, Hey1<br>capillary EC: Cdh5, Pecam1, Lpl<br>vein EC: Cdh5, Pecam1, Vwf, Hif1a, Icam1, Lrg1, Aplnr<br>endothelial, myelois cells: Cdh5, Pecam1, S100a8, S100a9, Csf1, Itgam (Walter)
	Myeloid	S100A8, S100A9, DEFA3,S100A12, LYZ (Yin)<br>CD14, F13A1, ITGAX, MS4A6A, FOLR2 (Lai)
	Adipocyte	ADIPOQ, PLIN1, LPL, EFNA5, MAML2 (Kedlian)<br>PPARG, PLIN1, AQP7, GPAM, ADIPOQ (Lai)<br>Acaca, Car3, Tenm4, Tmem45b, Pde3b, Prkar2b, Slc1a5, Pparg, Gm16168, Ctcflos, Tshr, Slc1a3, Fgf14, Negr1, 4933406I18Rik, Acsl1, Sik2, Nrg4, Lipe, Plin1, Fabp4, Fam13a, Cidec, Acvr1c, Adipor2, Fasn, Ankef1, Pck1, Slc36a2, Rgs7 (Sun)<br>Slc1a3, Fam13a, Car3, Ctcflos, Negr1, Nnat, Slc1a5, Nrg4, Pparg, Gm16168 (Cai)
	Pericytes	Acta2, Rgs5, Pdgfrb, Tagln, Myh11, Myl9, Abcc9, Kcnj8, Vtn, Gucy1a1, Mylk (Zhang)<br>ACTA2, RGS5, MYO1B, PDGFRB (Jing)<br>Gucy1a1 (Kimmel2)<br>Pericyte: Pecam1, Pdgfrb, Rgs5, Vtn, Kcnj8, Cspg4 (Peng)<br>RGS5, ABCC9, CPE, NOTCH3, IGFBP7 (Yin)<br>RGS5, ABCC9, ACTA2 (Kedlian)<br>MYO1B, STEAP4, EGFAM, KCNJ8, ACTA2, MYH11, TAGLN, MYL9 (Lai)<br>RGS5, NDUFA4L2, HIGD1B (Shen)<br>Trpc3, Cacna1c, Dlc1, Opcml, Kalrn, Lin7a, Plcl1, Myo1b, Rgs5, Pde8b, Slc35f1, Ano1, Gucy1a2, Cacnb2, Il34, Pdgfrb, Notch3, Pde3a, Prkg, Slc2, a3, Cald1, Eps8, Plxdc1, Abcc9, Sox5, Mast4, Adap2, Mrvi1, Gucy1a1, Sgip (Sun)<br>Cacna1c, Kalrn, Lin7a, Prkg1, Pde3a, Gucy1a2, Dlc1, Opcml, Myo1b, Rgs5 (Cai)<br>Rgs5 (Ma)<br>FLT1, RGS5 (Hettinger)<br>Ptprc, Cd52, Col3a1, Rgs5 (Lee)<br>Rgs5, Kcnj8, Vtn, Abcc9, Ndufa4l2 (Bi)<br>pericytes, smooth muscle cells: Rgs5, Acta2, Ednrb, Cspg4, Pdgfrb, Myl9, Myh11 (Walter)
	Neurons	Mpz, Mbp, Sox10, Gatrn, Pmp22, Mal, Drp2, Fxyd3, Plp1, S100b, Pry, Sdc4, Dag1, Kcna1, Dbi, Kcna2, Gpm6b (Zhang)<br>Ptprc, Cd52, Col3a1, Mpz (Lee)<br>Mpz, Mbp, Gatm, Pmp22, Fxyd3, Mal, Col20a1, Ptn, Lyz2, Pou3f1, Postn, Plp1, Gpm6b, Lgi4, Pdgfa (Bi)<br>**Neural cell:** Ptn, Gpm6b, Dbi (Peng)
	Tenocytes	Frnod, Thbs4, Tnc, Dcn, Comp, Chad, Angptl7, Prg4, Tnmd, Col1a1, Prelp, Col1a2, Col20a1, Ptn, Kcna1 (Zhang)<br>Tnmd (Kimmel2)

© 2025. Liu C et al. Published online at http://www.aginganddisease.org/EN/10.14336/AD.2025.0701

		Mkx, Col12a1, Edil3, Thbs4, Itgbl1, Nox4, Eya2, Abi3bp, Mylk (Petrany)<br>FMOD, TNMD, MKX (Kedlian)<br>Chad, Tnc (Liu L.)<br>Erbb4, Col22a1, Kcnh7, Fras1, Lama2, Prkg1, Adamts20, Limch1, Sgcd, Col24a1 (Cai)<br>TNMD (Hettinger)<br>Scx, Fmod, Thbs4, Angptl7, Comp, Col1a1 (Larouche)<br>Fmod, Thbs4, Comp, Angptl7, Chad, Tnmd, Tnc (Bi)<br>Col1a1, Dcn, Apod, Tnmd, Scx (Walter)
	Schwann cell	NCAM1, NRXN1, CDH19 (Jing)<br>MBP, PLP1, S100B, GFAP (Lai)<br>Plp1, Ncam1 (Liu L.)<br>mSchwann: MPZ, MBP, LPL<br>nmSchwann: L1CAM, LPL (Kedlian)<br>schwann, neural/glial cells: Ptn, Mpz (Walter)
	Tendon fibroblast	DCN, GSN, THBS4 (Jing)<br>Thbs4 (Ma)<br>Col3a1, Tnmd (Lee)<br>**Tendon:** Thbs4, Itgbl1, Nox4, Mylk, Edil3, Ust, P3h2, Ccbe1, Chodl, Col12a1, Myo1d, Lpar1, Antxr1, Piezo2, Eya2, Lrrk1, Tnc, Ccnd3, Aff3, Tshz2, Abi3bp, Kirrel3, Lhfp, Naaladl2, Ptprd, Mpp7, Nlgn1, Gpc6, Ntng1, Tenm2 (Sun)
	Fibroblast	Col1a1, Col1a2, Col3a1, Serpinf1, Fbln1, Dcn, Cxcl14, Smoc2, Fmod, Thbs4, Comp (Peng)<br>APOD, CFD, DCN, CXCL14, MGP (Yin)<br>Dcn (Kurland)<br>FB: PDGRFA, DCN<br>EnFB: DCN, ANGPTL7, OSR2<br>PnFB: DCN, NGFR, SLC22A3, ITGA6 (Kedlian)<br>Fibroblast-like: CD34, PTGDS, MYOC, STEAP4, TAGLN, FMOD, PDPN (Lai)<br>Fibrocytes: Cd34, Col1a1 (Sousa)
	Smooth muscle cell	MYH11, ACTA2 (Jing)<br>PDGFRB, CARMN, RGS5, CALD1, ACTA2 (Perez)<br>TAGLN, ACTA2, MYL9, MYH11, ADIRF (Yin)<br>MYH11, ACTA2, SORNS2 (Kedlian)<br>ACTA2, MYH11, TAGLN, MYL9, MYOCD (Lai)<br>Acta2, Myl9 (Liu L.)<br>Myh11, Dgkb, Acta2, Cacnb2, Cacna1c, Pde3a, Notch3, Flna, Thsd4, Rbpms, Cald1, Carmn, Sox5, Mylk, Ppm1e, Gucy1a1, Prkg1, Itga8, Ano1, Frmd4b, Sntb1, Mrvi1, Lmod1, Olfr78, Stac, Gpc6, Dlc1, Rgs6, Synpo2, Csmd (Sun)<br>Dgkb, Cacnb2, Myh11, Pde3a, Kcnab1, Prkg1, Cacna1c, Thsd4, Rcan2, Acta2 (Cai)<br>Myh11 (Ma)<br>MYH11 (Hettinger)<br>Rgs5 (Kurland)<br>Postn, Rgs5 (Oprescu)<br>Rgs5, Myl9, Acta2, Tagln, Tpm2 (Larouche)<br>Acta2, Tagln, Myh11, Myl9, Mylk (Bi)<br>SM1: Myh11, Dlc1, Kalrn, Cacna1c, Lin7a, Trpc3, Opcml, Pde3a, Il34, Cald1, Mast4, Plcb4, Ldlrad3, Ror1, Mylk, Cacnb2, Dgkb<br>SM2: Myh11, Slit3, Dlc1, Kalrn, Cacna1c, Lin7a, Trpc3, Opcml, Pde3a, Il34, Cald1, Mast4, Plcb4, Ldlrad3, Ror1, Mylk, Cacnb2, Dgkb, Mylk, Dgkg, Acta2, Pip5k1b, Ppm1e, Myh11, Cacnb2, Dgkb (Petrany)
	Immune cells	Ptprc, Cd52 (Lee)<br>Cd74 (Kurland)<br>Ptprc, Hbb-bs, , Hbb-bt, Hba-a1, S100a9, S100a8 (Larouche)<br>Ptprc, Mctp1, Acta1, Aldoa, Ccnd3 (Petrany)<br>PTPRC (Shen)<br>T/NK/Macrophages: CSF1R, NKG7, CD3E, STAB1, MS4A6A, CD74, LILRB5, IL10RA (Perez)<br>RBC: HBA1 (Kedlian)<br>Eosiniphill: EPX, PRG2 (Kedlian)<br>Eosinophils: Ly6c2, Cxcr2, Csf3r, S100a8, S100a9, Slglecf (Sousa)<br>Granulocytes: ITGAL, S100A9 (Hettinger)<br>Antigen presenting cell: CD7 (Hettinger)

© 2025. Liu C et al. Published online at http://www.aginganddisease.org/EN/10.14336/AD.2025.0701

	Erythrocyte	PPARG, PECAM1, FLT1, CDH5, CD34, VWF, JAM2, HBG2, HBE1 (Lai)<br>Pecam1, Vwf (Lee)<br>Hba-a1, Hba-a2, Hbb-bs, Hbb-bt (Walter)
	Dendritic cells	Ciita, Wdfy4, Htr7, Ctnnd2, Bcl11a, Cbfa2t3, Flt3, Gm15987, Grk3, Tbc1d8 (Cai)<br>cDC1: XCR1, CLEC9A<br>cDC2: CD1C, CLEC10A<br>pDC: CCR7, LILRA4, IL3RA, IRF7 (Kedlian)<br>Fscn1+ DC: Cxcl16, Fscn1, Cd74, H2-Aa, H2-Ab1, Cd83, Flt, Ccl5, Ccr7<br>Xcr1+ DC: Cd74, H2-Aa, H2-Ab1, Cd83, Flt, Xcr1, H2-Oa, H2-Ob<br>Cd72+ DC: Cd74, H2-Aa, H2-Ab1, Itgax, Cd72<br>Cd209a+ DC: Cd74, H2-Aa, H2-Ab1, Cd209a, Klrd1, H2-Oa (Walter)<br>DC-1: Cd209a<br>DC-2: Itgal, Flt3<br>DC-3: Flt3 (Sousa)
	Neutrophils	Retnlg, Itgam, Cxcr2, Ptprc, S100a9, S100a8, Lcn2, Il1b, Wfdc21, Slpi (Zhang)<br>FCGR3B, CSF3R, SORL1 (Kedlian)<br>S100a9, S100a8, Retnlg, Ngp, Il1b, Lcn2, Slpi (Bi)<br>Cxcr4, Csf1, S1000a8, S100a9, Sell, Cd14, Mmp9, Ptprc (Walter)<br>Ptprc, Itgam, Itgal, Cxcr2, Csf3r, S100a8, S100a9, Ly6g, Ngp (Sousa)
	Monocyte	CD14, S100A8, S100A12, CD163 (Kedlian)<br>Ptprc, Lyz2 (Liu L.)<br>Plcb1, Gda, Arhgap26, Pid1, Gpr141, Gm5150, Alcam, Fam49b, Tgfbi, Fgr (Cai)<br>ITGAL (Hettinger)
	Macrophage	Cd74, Cd68, Retnla, Lyz2, H2-Aa, H2-Eb1, H2-Ab1, C1qa, Pf4, C1qb, C1qc (Zhang)<br>CD163, CPM (Jing)<br>Cd74, H2-Aa, Ptprc, S100a8, S100a9, Lyz2, Lrg1 (Peng)<br>CD14, CD163, C1QA, LYVE1 (Kedlian)<br>Ptprc, Lyz2, Adgre1 (Liu L.)<br>F13a1, Mctp1, Rbpj, Mrc1, Slc9a9, Pid1, Dab2, Ptprj, Stab1, Frmd4b, Cd163, Dock2, Plekhg5, Inpp5d, Runx1, Arhgap15, Tbxas1, Pip4k2a, Zswim6, Fyb, Aoah, Ptprc, Iqgap2, Adgre1, Myo5a, Dock8, Cfh, Myo1f, Gas7, Zeb2 (Sun)<br>ADGRE1 (Hettinger)<br>Pan, pro-inflammatory mac: ADGRE1, AIF1, CCL2, CCL9, CCR2, CD68, CD86, CSF1R, IER3, Il1β, IRF5, MAFF, MARCKSL1, NFκBIZ, TLR2, TNF (Fix)<br>Cd68, Top2a (Oprescu)<br>Cd74, Retnla, Lyz2, C1qa, C1qb, C1qc, Fcer1g, Tyrobp, Ctss, Ccl6, Mrc1, Cd52, Ctsc, Laptm5 (Bi)<br>Cluster 0: Pf4, Folr2, Selenop, Lyve1, Hspa1a, Fxyd2, Jund, Gm42418<br>Cluster 1: Pf4, Folr2, Selenop, Lyve1, Hspa1a, Fxyd2, Etv3, Sdc4, Jund, Gm42418<br>Cluster 2: Lyz1, Fn1, Retnla, Lpl, Mmp19, Isg15<br>Cluster 3: Thbs1, Plac8, Chil3, Ly6c2, Tmsb10<br>Cluster 4: Gngt2, Itgal, Pglyrp1, Ace, Dusp16<br>Cluster 5: Bcl2a1b, Basp1, Cxcl2, Ccl3, Ccl4, Tnf,<br>Cluster 6: Spp1, Gpnmb, Fabp5, Trem2, Ctsd, Lgals3<br>Cluster 7: Etv3, Wnt11, Cd209a, Cd74, Tnip3, Nr4a3, Il1b<br>Cluster 8: Il1b, S100a9, S100a8, Retnlg, Hdc, Slpi, Il1r2<br>Cluster 9: Top2a, Pclaf, Ube2c, Stmn1, Tubb5, Hmgb2<br>Cluster 10: Fabp4, Adamts1, Tm4sf1, Igfbp7, Sparc, Gng11 (Krasniewski)<br>Arg1+Mac: Hmox1, Osbpl8, Gabra2, Rbm47, Pid1, F13a1, Spp1, Mrc1, Dab2, Kcnk13<br>Mac1: Lgmn, Rbm47, Apobec1, Gpnmb, Fyb, St18, Myo1f, Alcam, Ctss, Lyn<br>Mac2: Rbpj, F13a1, Mrc1, Dab2, Frmd4b, Hdac9, Slc9a9, Mctp1, Plekhg5, Stab1 (Cai)<br>Perivascular macrophage: Cd163 (Ma)<br>cycling Mono/macro: Smc4, Cdk1, Hmgb2<br>Cxcl10+ Mono/macro: Cxcl10, Ctss<br>patrolling Mono/macro: Ctsa, Ctsb, Ctsl, Ctsz, Fabp5<br>Ccr2+ Mono/macro: Ly6c2, Ccr2, Ccl9, Ccl6, Tgfbi<br>Mrc1+ Mono/macro: Ccl9, Ccl6, Cd163, Mrc1, C1qa<br>Cx3cr1+ Mono/macro: Cx3cr1, C1qa, Ctss (Walter)<br>mono/macs 1: Adgre1, Cd68, Cx3ce1<br>mono/macs 2: Itgam, Cd68, Ly6c2<br>mono/macs 3: Adgre1, Mrc1, Lyve1<br>mono/macs 4: Ptprc, Itgal, Adgre4<br>proliferating mono/macs: Cd68, Mkl67, Cdk1 (Sousa)

© 2025. Liu C et al. Published online at http://www.aginganddisease.org/EN/10.14336/AD.2025.0701

		Subpopulation:<br>LYVE1+/MHCIIlo: Lyve1, Folr2, Cd163, Mrc1<br>LYVE1+/MHCIIhi: Lyve1, Folr2, Cd163, Mrc1, H2-Eb1, H2-Ab1<br>LYVE1−/MHCIIhi: H2-Eb1, H2-Ab1<br>LYVE1−/MHCIIlo: Gsr, Fabp4 (Krasniewski)<br>Cluster 0: C3ar1, Pmp22, Lmna, Cd14, Cxcl2, Ccl7, Ccl2, Hmox1, Ccl3, Ccl4, C1qa, Sepp1, Folr2, Apoe<br>Cluster 1: Lsp1, Ear2, Tnip3, Ramp3, Ccr7, Cd209a, Rernla, Fn1<br>Cluster 2: C1qa, Sepp1, Rhob, Mafb, Folr2, Apoe, Jun, Klf2, Hspa1a<br>Cluster 3: Apoc2, Plac8, Hp, Chi3l3, Ly6c2, Ms4a4c, Gngt2, Clec4e, Thbs1<br>Cluster 4: Plac9, Col1a2, Col1a1, Col3a1, Dcn, Gsn, Gpx3, Mgp, Thbs4, Fmod, Igfbp7<br>Cluster 5: Cdh5, Cav1, Gpihbp1, Fabp4, Gng11, Aqp1, Id1, Tm4sf1, Ly6c1, Sdpr, Igfbp7, Pim3<br>Cluster 6: Cst3, Plbd1, Cd24a, Ckb, Tmsb10, Irf8<br>Cluster 7: Hp, Chi3l3, Ly6c2, Clec4e, Thbs1, 1100001G20Rik, Retnlg, Lcn2, Ltf, Pglyrp1, Ngp, Camp, S100a8, S100a9, Ifitm1<br>Cluster 8: Cdh5, Cav1, Gpihbp1, Fabp4, Gng11, Aqp1, Id1, Tm4sf1, Ly6c1, Sdpr, Igfbp7, Pim3, Cd79a, Ly6d, Cd79b, Sdpr, Igfbp7, Pim3 (Liu)<br>M2-like: CD163<br>M1-like: C1QA, C1QB, C1QC (Shen)
	Mast cell	TPSB2, HPGD, CTSG, TPSAB1, CPA3 (Yin)<br>TPSB2, MS4A2 (Kedlian)<br>TPSB2, MS4A2, CTSG, CD69 (Lai)<br>MCPT1l1 (Hettinger)<br>TPSAB1, MS4A2, KIT, CPA3 (Shen)
	Lymphoid cell	CCL5, CD3D, NKG7, GZMA, TRBC2 (Yin)<br>ILC2s: Il1rl1, Il5 (Sousa)<br>Lymphocyte: CCL4, CXCR4, IL7R, CD3D, CD69 (Lai)
	T cell	SKAP1, TOX (Jing)<br>Cd3d (Kimmel2)<br>PTPRC, CD3D, IL7R (Kedlian)<br>Ptpre, Cd3g (Liu L.)<br>Bank1, Dock2, Ptprc, Gm2682, Skap1, Aff3, Prkcb, Ikzf1, Ccnd3, Pax5, Grap2, Ralgps2, Dock10, Arhgap45, Gm15987, Syk, Nedd, Ikzf3, Tmem163, Bcl11a, Scml4<br>Dock8, Elmo1, Arhgap15, Runx1, Ripor2, Themis, Itga4, Arhgef18, Bach2 (Sun)<br>Skap1, Gm2682, Bcl2, St6galnac3, Grap2, Tox, Cd247, Camk4, Ripor2, Bcl11b (Cai)<br>CD3e, NKG7 (Hettinger)<br>Ptprc, Cd52, Cd3d (Lee)<br>Cd3e, Cd4 (Sousa)<br>non-cycling T cells: Cd8a, Cd8b1, Cd3e<br>cycling T cells: Cd8a, Cd8b1, Cd3e, Smc4, Cdk1, Hmgb2<br>Cd4+ T cells: Cd8a, Cd8b1, Flt3, Sell, Cd4, Ly6c2 (Walter)
	B cell	Igkc, Iglc1, Ighm, Cd79a, Cd74, Cd79b (Bi)<br>Cd19, Cd22, Ms4a1 (Walter)<br>Ptprc, Cd19 (Liu L.)<br>CD79A, TCL1A;<br>B-plasma: MZB1, JCHAIN (Kedlian)<br>Plasma B: MZB1, CD79A, IGHG1, IGHG4. IGHG3 (Yin)<br>B cells 1: Cd79a, Cd19<br>B cells 2: Ly6c2, Mkl67, Cd79a (Sousa)
	NK cell	NKG7, PRF1, FCGR3A (Kedlian)<br>NKG7 (Hettinger)<br>CD247, NKG7 (Shen)<br>Nkg7, Gzma, Klra4, Klre1 (Walter)<br>Klrb1c, Ncr1 (Sousa)

© 2025. Liu C et al. Published online at http://www.aginganddisease.org/EN/10.14336/AD.2025.0701

SUPPLEMENTARY DATA

Supplementary Table 6. Abbreviation list

**Full name of abbreviation in the manuscript**	
ADAM28: ADAM Metallopeptidase Domain 28	Myf5: Myogenic Factor 5
Akt: Protein kinase B	Myh2: Myosin Heavy Chain 2
AP-1: Activator Protein 1	Myh7: Myosin Heavy Chain 7
Apoe: Apolipoprotein E	Mylk2: Myosin Light Chain Kinase 2
Arntl: Aryl Hydrocarbon Receptor Nuclear Translocator-Like Protein 1	Myod: Myogenic Differentiation
Atf3: Activating Transcription Factor 3	Myog: Myogenin
Bdnf: Brain Derived Neurotrophic Factor	NCR1: Natural Cytotoxicity Triggering Receptor 1
Ccl: C-C Motif Chemokine Ligand	Nfe2l2: Nuclear Factor Erythroid 2-Related Factor 2
Ccn2: Cellular Communication Network Factor 2	Nfkb1: Nuclear Factor NF-Kappa-B P105 Subunit
CDC42 GTPase Cycle: Cell Division Cycle 42 Guanosine Triphosphate Cycle	NF-κB: Nuclear Factor Kappa B
Cdkn: Cyclin Dependent Kinase Inhibitor	Nlrp3: NLR Family Pyrin Domain Containing 3
Cebpb: CCAAT Enhancer Binding Protein Beta	NMJ: Neuromuscular Junction
Clock: Clock Circadian Regulator	non-G1: Non-Gap 1 Phase
Col1a1: Collagen Type I Alpha 1 Chain	Notch: Neurogenic Locus Notch Homolog Protein
CR: Caloric Restriction	Nr1h3: Nuclear Receptor Subfamily 1 Group H Member 3
Ctsa: Cathepsin A	Nr4a3: Nuclear Receptor Subfamily 4 Group A Member 3
Cxcl: C-X-C Motif Chemokine Ligand	Nrap: Nebulin Related Anchoring Protein
DCLK1: Doublecortin Like Kinase 1	OTUD1: OTU Deubiquitinase 1
DCs: Dendritic Cells	OXPHOS: Oxidative Phosphorylation
DEGs: Differentially Expressed Genes	p16: Cyclin Dependent Kinase Inhibitor 2A
Dpi: Days Post Injury	p53: Tumor Protein P53
ECM: Extracellular Matrix	Pax: Paired Box
ECs: Endothelial Cells	Pdk4: Pyruvate Dehydrogenase Kinase 4
Enah: ENAH Actin Regulator	PI3K-Akt: Phosphatidylinositol 3-kinase /protein kinase B
ENOX1: Ecto-NOX Disulfide-Thiol Exchanger 1	Plin2: Perilipin 2
ERK1/2: Mitogen-activated protein kinase 1/2	PMF: Postsynaptic Muscle Fiber
Fabp: Fatty Acid Binding Protein	PPAR: Peroxisome Proliferator Activated Receptor
FAM189A2: Family with Sequence Similarity 189 Member A2	Ppargc1a: Peroxisome Proliferator-activated Receptor Gamma Coactivator-1 Alpha
FAP: Fibro-Adipogenic Progenitor	PRISMA: Preferred Reporting Items for Systematic Reviews and Meta-Analyses
Fbxo32: F-Box Protein 32	Prkag3: Protein Kinase AMP-Activated Non-Catalytic Subunit Gamma 3
FDA: Food and Drug Administration	Ras: Rat Sarcoma
Flnc: Filamin C	Runx1: RUNX Family Transcription Factor 1
Folr2: Folate Receptor Beta	S/G2/M: Synthesis Phase/Gap 2 Phase/ Mitosis Phase
FOXO: Forkhead Box O	S100: S100 Calcium Binding Protein
GBPs: Guanylate-Binding Proteins	SAA2: Serum Amyloid A2
Gngt2: G Protein Subunit Gamma Transducin 2	SAMP6: Senescence-Accelerated Mouse Strain P6
Gpnmb: Glycoprotein Non-Metastatic Protein B	scRNA-seq: Single-cell Ribonucleic Acid Sequencing
Hdac4: Histone Deacetylase 4	Sesn1: Sestrin 1
HLA: Histocompatibility Complex	Smad3: Mothers Against Decapentaplegic Homolog 3
ID1: Inhibitor of DNA Binding 1	SMCs: Smooth Muscle Cells
IFNγ: Interferon Gamma	snRNA-seq: Single-nucleus Ribonucleic Acid Sequencing
Igf1: Insulin Like Growth Factor 1	Sod1: Superoxide Dismutase 1
Igfn1: Immunoglobulin Like and Fibronectin Type III Domain Containing 1	Sox11: SRY-Box Transcription Factor 11
Ighm: Immunoglobulin Heavy Constant Mu	Spp1: Secreted Phosphoprotein 1
Il10: Interleukin 10	Spry1: Protein Sprouty Homolog 1
Il1b: Interleukin 1 Beta	TFs: Transcription Factors
IL-6: Inflammatory Cytokine Interleukin-6	Tgfb2: Transforming Growth Factor Beta 2
KLRD1: Killer Cell Lectin Like Receptor D1	TGFβ: Transforming Growth Factor β
Lox: Lysyl Oxidase	Th1-like effector memory CD4+ T cells: Th1-Lineage Transcription Pattern Effector Memory Helper T Cells
Ly6c2: Lymphocyte Antigen 6C2	Th2-like effector memory CD4+ T cells: Th2-Lineage Transcription Pattern Effector Memory Helper T Cells
Lyve: Lymphatic Vessel Endothelial Hyaluronan Receptor	Timp1: Tissue Inhibitor of Metalloprotease-1
Lyz1: Lysozyme 1	Tnfrsf12a: TNF Receptor Superfamily Member 12A

© 2025. Liu C et al. Published online at http://www.aginganddisease.org/EN/10.14336/AD.2025.0701

SUPPLEMENTARY DATA

M2 macrophage: Alternatively Activated Macrophages
MAPK: Mitogen-Activated Protein Kinase
MHC II: Major Histocompatibility Complex II
MME: Membrane Metalloendopeptidase
Mrc1: Mannose Receptor C-Type 1
mTORC1: Mammalian Target of Rapamycin Complex 1
MuSCs: Muscle Stem Cells
Musk: Muscle Associated Receptor Tyrosine Kinase
TNF-α: Tumor Necrosis Factor α
Tnnt: Troponin T
TREM2: Triggering Receptor Expressed on Myeloid Cells 2
Trim63: Tripartite Motif Containing 63
Vegfa: Vascular Endothelial Growth Factor A
Wnt: Wingless-Related Integration Site
γδ T cells: Gamma delta T cells

© 2025. Liu C et al. Published online at http://www.aginganddisease.org/EN/10.14336/AD.2025.0701
